# Lower respiratory tract co-infection of *Streptococcus pneumoniae* and respiratory syncytial virus shapes microbial landscape and clinical outcomes in children

**DOI:** 10.3389/fcimb.2025.1593053

**Published:** 2025-06-09

**Authors:** Xiaoran Yu, Xuemei Yang, Yiqin Song, Jie Yu, Tingting Jiang, He Tang, Xiaoxuan Yang, Xi Zeng, Jing Bi, Adong Shen, Lin Sun

**Affiliations:** ^1^ Laboratory of Respiratory Diseases, Beijing Pediatric Research Institute, Beijing Children’s Hospital, Capital Medical University, National Clinical Research Center for Respiratory Diseases, National Center for Children’s Health, Beijing, China; ^2^ Institute of Child and Adolescent Health, School of Public Health, Peking University, Beijing, China; ^3^ Baoding Hospital of Beijing Children’s Hospital, Capital Medical University, Baoding, China; ^4^ Henan International Joint Laboratory of Children’s Infectious Diseases, Children’s Hospital Affiliated to Zhengzhou University, Zhengzhou, China

**Keywords:** lower respiratory tract infections, *Streptococcus pneumoniae*, respiratory syncytial virus, microbiota, children

## Abstract

**Background:**

Lower respiratory tract infections (LRTI), which are most commonly caused by *Streptococcus pneumoniae* (SP) and respiratory syncytial virus (RSV), pose a substantial global health burden in children. However, the causal pathways of bacterial-viral co-infections, particularly the mechanisms by which commensal microbiota could modulate SP-RSV-associated LRTI outcomes remain to be elucidated.

**Methods:**

A population-based cross-sectional study was conducted on children aged 0–18 years who were admitted to Beijing Children’s Hospital and Baoding Children’s Hospital in China from September 2021 to August 2022. Children with LRTI who underwent respiratory pathogen testing were divided into SP single infection and SP-RSV co-infection groups, with sex- and time-matched non-LRTI children as controls. Sputum and LRT secretion samples were collected for microbiota analysis using 16S rRNA sequencing, and child characteristics were obtained from medical records and pharmacy data.

**Results:**

A total of 125 children with LRTI (84 with SP infection and 41 with SP-RSV co-infection) and 87 children without LRTI were recruited for this study. We found that LRT microbiota composition was strongly related to age, with a more pronounced increase in Shannon index within the first 5 years of life. Children with SP and RSV infection exhibited significantly altered microbiota composition in comparison to children without LRTI, particularly a higher abundance of *Streptococcus*. The competitive interactions among respiratory bacteria were found to be more complex in the SP single-infection group and simpler in the SP-RSV co-infection group.

**Conclusion:**

Our findings show that RSV co-infection exacerbates SP-induced LRTI microbiota disorder and disease severity. This study may help us to better understand the characteristics of SP-RSV interaction and provide direction for the pathogen diagnosis of LRTI.

## Introduction

Lower respiratory tract infection (LRTI) —including pneumonia and bronchiolitis—is a leading cause of mortality and morbidity worldwide, resulting in an estimated 344 million incident episodes and 2.18 million deaths in 2021, with nearly a quarter of deaths occurring in children younger than 5 years (Bender et al., 2024). Concurrently, the rising prevalence of antimicrobial resistance and the COVID-19 pandemic have further emphasized the role of respiratory pathogens in the etiology of severe pneumonia (Li et al., 2020; Dagan et al., 2023), and raised the concern that bacterial-viral co-infection may cause significant complications leading to increased morbidity and adverse prognosis (Feldman and Anderson, 2021). Therefore, it is crucial to study the primary causes of bacterial-viral co-infection and factors modulating susceptibility and the clinical outcomes of LRTI.

*Streptococcus pneumoniae* (SP) and respiratory syncytial virus (RSV) are two of the leading pathogens of childhood community-acquired pneumonia (Liu et al., 2023; Li et al., 2024). The relevance of their interaction in modulating severe disease has gradually gained attention based on the strong seasonal epidemiological association between these two pathogens, and the association between pneumococcal vaccination and the reduced RSV hospitalizations in children (Besteman et al., 2024). As demonstrated in several previous studies, RSV infection has been shown to promote pneumococcal adherence to human airway epithelial cells through two principal mechanisms: (1) enhancing bacterial binding to epithelial cells through modulation of adhesion molecules and bacterial virulence gene upregulation; and (2) impairing the host immune response (Hament et al., 2005; Avadhanula et al., 2006; Smith et al., 2014). It has been demonstrated that certain pneumococcal serotypes (e.g., 8, 15A, and 19F) enhance RSV replication *in vitro*, suggesting that there are strain-specific interactions between SP and RSV (Besteman et al., 2024). In addition, several studies have revealed multidirectional interplays between the host immune system, bacteria, and viruses, which complicates the elucidation of SP and RSV co-infections (Besteman et al., 2024). Immunological studies in humans with SP and RSV co-infections have revealed the activation of a specific subset of anti-inflammatory neutrophils. These cells have the potential to suppress T cells and thus help a viral infection (Pillay et al., 2012; Cortjens et al., 2017). Further investigation is required to better understand the multidirectional interaction between these two pathogens and the respiratory microbiome to inform and enhance healthcare practices.

Similar to the essential role played by the gastrointestinal microbiome in maintaining homeostasis and overall health, the composition of the commensal microbiome of the respiratory tract has been demonstrated to modulate the host immune response, thereby potentially influencing susceptibility to bacterial or viral respiratory infections (Man et al., 2019; de Steenhuijsen Piters et al., 2022). The normal composition of bacteria, fungi, and viruses in the respiratory tract is considered to be beneficial to human health (Li et al., 2024). Disruption to the “colonization” equilibrium of the standard composition may result in the prevalence of potential pathogens, which can progress to LRTI and even sepsis (Natalini et al., 2023). *Streptococcus*, a constituent of the normal respiratory tract microbiome, is carried asymptomatically in children. Nevertheless, its colonization can become pathogenic, with evidence suggesting that RSV is a common trigger of such a transition (Man et al., 2017). Despite the advances made in the field of respiratory microbiota, the causal pathways of bacterial and viral co-infections, especially the mechanisms by which commensal microbiota could modulate SP-RSV-associated LRTI outcomes remain largely to be elucidated (de Steenhuijsen Piters et al., 2020).

In this study, we conducted a population-based LRT microbiome survey in 125 LRTI children with SP single infection or SP-RSV co-infection and recruited 87 without respiratory infections as controls. We aimed to 1) characterize the lower respiratory microbiota of the children aged 0−18 years; 2) elucidate the potential causative role of lower respiratory microbiota in modulating SP single infection and the inducement of RSV in SP-associated LRTI; 3) establish a simple microbial classifier based on a machine learning model to distinguish different LRTI types.

## Methods

### Study participants

This study was conducted at Beijing Children’s Hospital and Baoding Children’s Hospital in China between September 2021 and August 2022. Children diagnosed with LRTI who underwent respiratory pathogen testing were included. Inclusion criteria included: (1) the presence of clinical symptoms such as fever, cough, or wheezing; and (2) typical radiographic evidence of pneumonia or bronchiolitis, such as alveolar or interstitial lung infiltration; and (3) positive molecular identification of SP and/or RSV, and exclusion of other pathogenic infections; and (4) had complete clinical data and blood routine examination data. Children with symptom onset longer than five days were excluded to minimize the potential confounding effect of disease duration on results. Children diagnosed with LRTI were assigned to either the SP single infection or the SP-RSV co-infection group. A case was classified as severe if any of the following conditions were met: (1) hypoxemia; or (2) imaging findings showing infiltration involving more than two-thirds of a unilateral lung, multi-lobar infiltration, pleural effusion, pneumothorax, lung atelectasis, lung necrosis, or lung abscess; or (3) the presence of extrapulmonary complications (The Subspecialty Group of Respiratory et al., 2024). According to clinical records and parental reports, none of the participants had received RSV passive immunization or pneumococcal conjugate vaccine.

The LRT secretions from children undergoing surgical operations were selected to represent healthy respiratory microbiomes if they met the following three criteria: (1) normal blood counts, C-reactive protein (CRP), and procalcitonin (PCT) levels at enrollment; and (2) no history of respiratory infections and had not received antibiotics in the past three months, and (3) physical examinations and chest radiographs confirming the absence of LRTI.

### Sputum and LRT secretions collection and pathogen detection

Respiratory specimens were collected on the day of admission. Prior to sample collection, each patient was required to rinse their mouth with sterile saline. In the case group, older children capable of expectoration were instructed to perform a deep cough to produce sputum samples in the early morning, using sterile containers for collection. For younger children or those unable to produce adequate sputum, induced sputum samples were obtained by inhalation of aerosolized 5% NaCl solution for 10–15 minutes, followed by deep coughing and collection of sputum plugs. The quality of the samples was then assessed using the Murray-Washington criteria: only samples containing fewer than 10 squamous epithelial cells and more than 25 leukocytes per low-power field were to be accepted for further processing. In the control group, LRT secretions were collected via routine suction after tracheal intubation during surgery. All specimens were immediately stored at -80°C for subsequent analysis.

Nucleic acids were extracted from the samples collected from patients and were semi-quantitative amplified using a multiplex PCR testing for the most common 15 virus and 14 bacteria. Furthermore, the simultaneous execution of SP culture was conducted on the collected samples.

### DNA isolation and amplification

Bacterial genomic DNA (including negative control samples) was isolated from quality-controlled sputum/respiratory secretions using the Qiagen Gel Extraction Kit (Qiagen, Hilden, Germany), with additional detection of the integrity of the extracted genomic DNA steps using 1% agarose gels. The V3−V4 hypervariable region of bacterial 16S rRNA gene was amplified using primers 341F (5′-CCTAYGGGRBGCASCAG-3′/5′-CCTACGGGNGGCWGCAG-3′) and 806R (5′-GGACTACNNGGGTATCTAAT-3′/5′-GGACTACHVGGGTWTCTAAT-3′) to generate sequencing library. All PCR reactions were carried out with 15 µL of Phusion^®^ High -Fidelity PCR Master Mix, 0.2 µM of forward and reverse primers, and about 10 ng template DNA. Thermal cycling conditions included an initial denaturation at 98°C for 1 min, followed by 30 cycles of denaturation at 98°C for 10 s, annealing at 50°C for 30 s, extension at 72°C for 30 s, and a final extension at 72°C for 5 min. Equal volumes of 1X loading buffer containing SYBR Green were mixed with PCR products, followed by electrophoresis on a 2% agarose gel for detection. The PCR products were then combined in equimolar ratios, and the pooled products were purified using the Universal DNA Purification Kit (TianGen, China; Catalog #: DP214). Sequencing libraries were generated using NEB Next^®^ Ultra™ II FS DNA PCR-free Library Prep Kit (New England Biolabs, USA, Catalog #: E7430L) following manufacturer’s recommendations and indexes were added, then the final libraries were sequenced on the Illumina NovaSeq 6000 platform with 250bp paired-end reads generated.

### Taxonomic assignment

Paired-end reads were assigned to samples based on their unique barcode and truncated by cutting off the barcode and primer sequence and then merged using FLASH (Version 1.2.11, http://ccb.jhu.edu/software/FLASH/) (Magoč and Salzberg, 2011). Quality filtering on the raw tags was performed using the Fastp (Version 0.23.1) software to obtain high-quality Clean Tags (Bokulich et al., 2013). The tags were compared with the Silva 138.1 database (https://www.arb-silva.de/) using UCHIME Algorithm (http://www.drive5.com/usearch/manual/uchime_algo.html) to detect chimera sequences, then the chimera sequences were removed and the effective tags were obtained (Edgar et al., 2011).

Raw effective tags were denoised using tools available in QIIME2 software (Version 202006) with a Divisive Amplicon Denoising Algorithm (DADA) 2-based pipeline to obtain initial Amplicon Sequence Variants (ASVs) and feature table (Wang et al., 2021). Species annotation was performed using QIIME2 software and then classified against SILVA 138.1 database. Finally, the absolute abundance of ASVs was normalized using a standard of sequence number corresponding to the sample with the least sequences.

### Statistical analysis

#### Microbial diversity and composition

We used the “microeco” package to calculate alpha-diversity indices and assess the microbial diversity in different groups. Using the “segmented” package, we assessed the trend of the Shannon index across different ages in non-LRTI children and identified breakpoints in the 0−18 age range. The Davis test was used to evaluate the significance of the difference in slopes before and after a given breakpoint. The beta-diversity dissimilarity matrix was generated and tested by permutational multivariate analysis of variance (PERMANOVA) with 999 permutations using the “vegan” package. Principal coordinates analysis (PCoA) plots were then constructed to visualize the differences in microbial community composition. To evaluate the effects of respiratory infection-associated host and clinical factors on microbiota profiles, we conducted variation partitioning analysis based on redundancy analysis and explained by potential determinants through PERMANOVA in “vegan”. MetaStats analysis was conducted between paired groups to determine significantly different relative abundances of ASVs/genera, identified by Benjamini-Hochberg (BH)-adjusted *P* values <0.05 and a log2-fold change (FC) >1.0. Average relative abundance of the dominant phyla/genera found in the microbiota of the respiratory visualized by stacked bar plots using “ggplot2” package.

#### Microbial interaction network

Mantel tests were used to explore the potential link between the top-20 genera in abundance and age in healthy children, with correlations identified by Spearman’s correlation analysis using “linkET” package. Co-occurrence relationships among the top-10 genera in children with SP single infection and SP-RSV co-infection groups were analyzed using Spearman’s correlation and visualized using the “igraph” package.

#### Random Forest analysis

Random Forest regression was used to predict respiratory infections based on bacterial abundances using the “randomForest” package. First, we trained machine learning models on a randomly selected training set (80%), and subsequently applied to a withheld test set (20%) to assess performance from 125 LRTI children. Subsequently, we optimized four hyperparameters (e.g., mtry, ntree, nodesize, maxnodes) based on model performance in the test set to prevent overfitting. Finally, we repeated the random cohort split 10 times with the best hyperparameter combination to generate a distribution of random forest predictions on the test set. The receiver operating characteristic curve (ROC) for the categorical group was plotted at the genus level and the area under the curve (AUC) was calculated using the “pROC” package for result visualization. The optimum random forest model was chosen from 10-fold cross-validation, and genera with a significant Mean Decrease Accuracy were identified as important biomarkers.

The logistic regression model adjusted for age was used to analyze the risk of other clinical comorbidities in children with SP-RSV co-infection group and presented in a forest plot using the “forestplot” package. All analyses were performed and visualized in R version 4.3.1 within the RStudio Server version 2023.06.2. All statistical tests were two-sided, and *P* values were corrected for multiple testing using the BH method, with <0.05 considered to be significant.

## Results

### Demographic characteristics of the participants with LRTI

**Figure 1:**
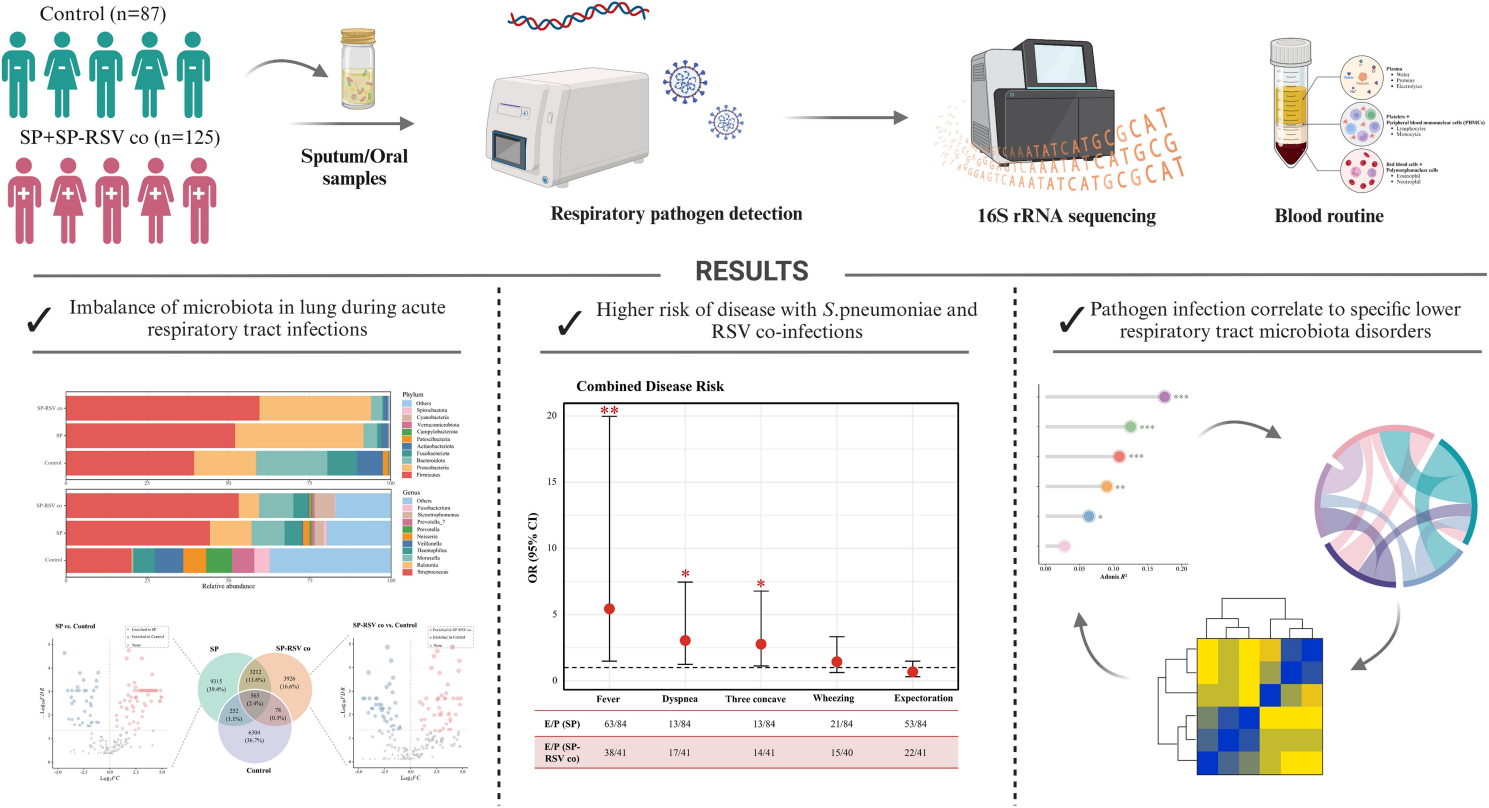
Experimental design of the study, analysis, and workflow schematic.

We characterized the LRT microbiota—sputum or respiratory secretions—based on 212 children in a cross-sectional survey, which included 125 LRTI children with 84 SP single infection and 41 SP-RSV co-infection, and 87 non-LRTI children aged 0−18 years. **Figure 1** provides an overview of the study workflow. The demographics and clinical characteristics are summarized in **Table 1**. A higher prevalence of fever, cough, dyspnea, and three depression sign of clinical symptoms was observed in children with SP-RSV co-infection compared to those with SP single infection. A higher proportion of children with severe disease was also observed in the SP-RSV co-infection group (all *P <*0.05, **Table 1**).

**Table 1:** Demographic and clinical characteristics of children stratified by the presence/absence of lower respiratory tract infection^a^.

Characteristics	LRTI (N=125)			Non-LRTI (N=87)	*P* value^†^
	SP si (N=84)	SP-RSV co (N=41)	*P* value^*^		
Age	3.0 (1.0, 4.0)	1.0 (0.5, 3.0)	0.005	7.3 (3.0, 14.8)	<0.001
Sex			0.32		0.47
Female	36 (42.9)	13 (31.7)		29 (33.3)	
Male	48 (57.1)	28 (68.3)		58 (66.7)	
Disease Severity			0.03	−	
Mild	66 (78.6)	24 (58.5)		−	−
Severe	18 (21.4)	17 (41.5)		−	−
LRTI phenotype			0.003		
Pneumonia	56 (66.7)	38 (92.7)		−	−
Bronchiolitis	28 (33.3)	3 (7.3)		−	−
Clinical symptoms					
Fever	63 (75.0)	38 (92.7)	0.03	−	−
Cough	71 (84.5)	41 (100.0)	0.005	−	−
Expectoration	53 (63.1)	22 (53.7)	0.41	−	−
Wheezing	21 (25.0)	15 (36.6)	0.22	−	−
Dyspnea	13 (15.5)	17 (41.5)	0.003	−	−
Three depressions sign^b^	13 (15.5)	16 (39.0)	0.007	−	−
Laboratory findings on admission					
White blood cell count (10^9^/L)	7.4 (5.6, 10.1)	8.1 (6.8, 9.4)	0.52	−	−
Platelets count (10^9^/L)	339.0 (255.3, 445.8)	380.5 (306.8, 452.8)	0.46	−	−
Neutrophil percentage (%)	38.8 (28.7, 50.8)	38.7 (30.9, 55.9)	0.85	−	−
Lymphocyte percentage (%)	47.6 (35.0, 60.9)	52.0 (33.8, 59.9)	0.75	−	−
Monocyte percentage (%)	8.4 (7.2, 9.8)	8.1 (6.6, 10.1)	0.37	−	−
Eosinophil percentage (%)	1.3 (0.4, 3.3)	1.4 (0.3, 2.5)	0.56	−	−
Basophil percentage (%)	0.2 (0.2,0.4)	0.3 (0.2, 0.3)	0.94	−	−
Absolute neutrophil count (10^9^/L)	2.9 (1.5, 4.6)	3.3 (2.2, 4.8)	0.46	−	−
Absolute lymphocytes count (10^9^/L)	3.2 (2.4, 4.3)	3.9 (2.5, 4.7)	0.32	−	−
Absolute monocytes count (10^9^/L)	0.6 (0.4, 0.9)	0.6 (0.5, 0.8)	0.77	−	−
Absolute eosinophils count (10^9^/L)	0.11 (0.03, 0.23)	0.11 (0.02, 0.20)	0.64	−	
Absolute basophil count (10^9^/L)	0.02 (0.01, 0.03)	0.02 (0.01, 0.03)	0.78	−	−

a Values are presented as median (interquartile range) or n (%);

b Inward depression of the suprasternal fossa, supraclavicular fossa and intercostal space.

LRTI, Lower respiratory tract infection; SP si,*Streptococcus pneumoniae* single infection; SP-RSV co, Streptococcus pneumoniae-respiratory syncytial virus co-infection;

*P values for comparing SP single infection and SP-RSV co-infection groups; †P value for comparing LRTI and non-LRTI groups.

### The LRT microbiota across ages in children

**Figure 2:**
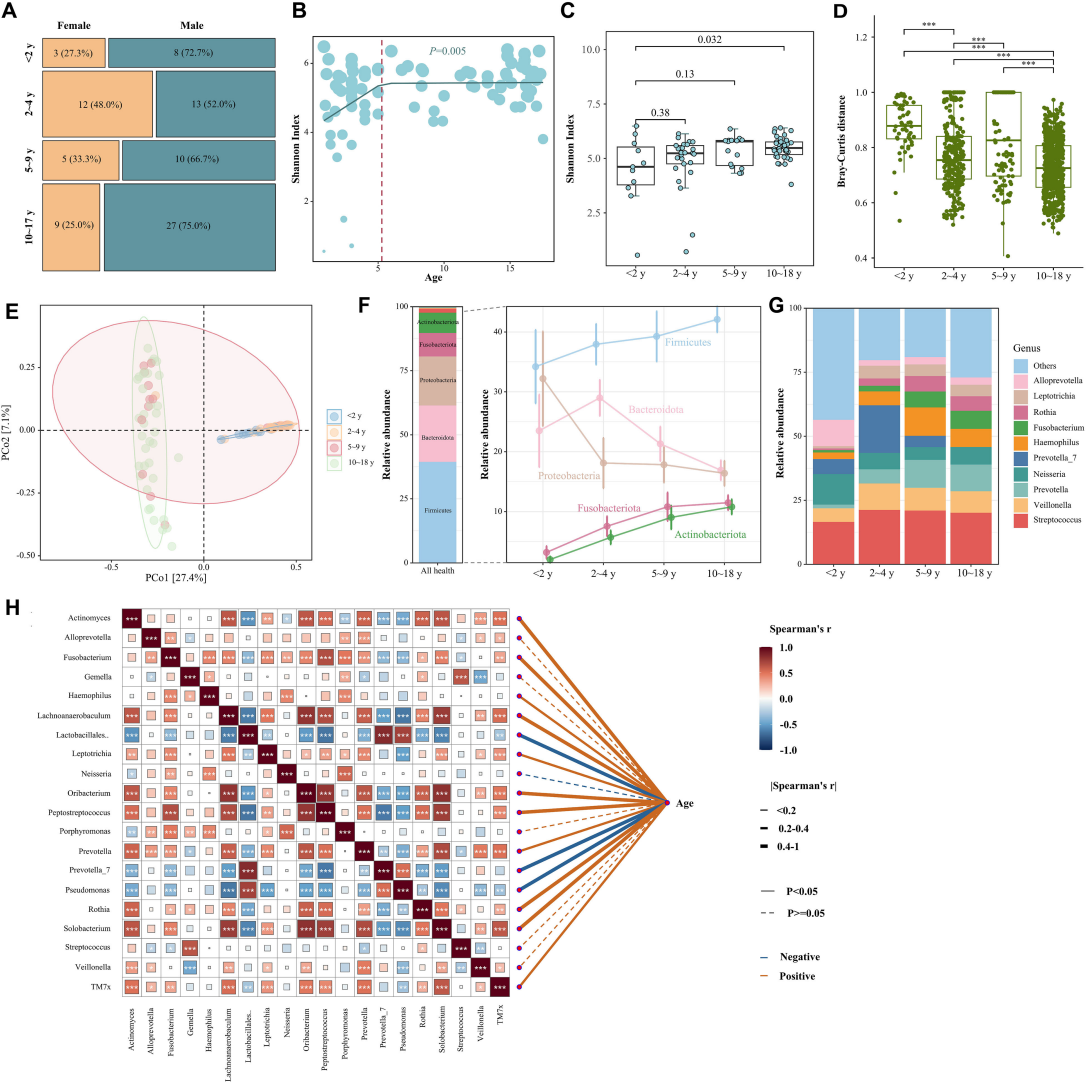
The lower respiratory tract microbiota across ages in healthy children. **(A)** Mosaic plot of the proportion of participants in each respiratory secretions microbial cluster per age and sex category. The varying width of each age group represents the proportion of the study population within that specific age group. **(B)** ASV-level Shannon diversity across age in years in different age. Through piecewise regression analysis and the Davies test, significant breakpoints (denoted by red dashed lines) were identified, indicating shifts in Shannon diversity at specific age points. **(C)** Shannon diversity across different age groups. *P* values were calculated using Wilcoxon rank sum test. **(D)** Bray-Curtis dissimilarities between samples per niche in individuals aged under 18 years. Boxplots represent the 25th and 75th percentiles (lower and upper boundaries of boxes, respectively), the median (middle horizontal line), and measurements that fall within 1.5 times the interquartile range (IQR; distance between the 25th and 75th percentiles; whiskers). *P* values were calculated using Wilcoxon rank sum test. **(E)** Principal coordinate analysis (PCoA) based on Bray-Curtis dissimilarities of different age groups. Explained variance (R2) and *P* values were calculated using PERMANOVA tests. **(F)** Average relative abundance of the dominant phylum found in the microbiota of the respiratory represented by stacked bar plots. The right line chart showed the abundance change of phylum along different age groups. **(G)** Average relative abundance of the ten most frequent genera found in the microbiota of the respiratory represented by stacked bar plots. **(H)** Analysis of the spearman correlations between the twenty most frequent genera found in the microbiota of the respiratory and age in children. *p < 0.05, **p < 0.01, ***p < 0.001.

As illustrated in **Figure 2A**, the composition characteristics of non-LRTI children were stratified by sex and age. Using piecewise regression model and Davies test, we observed a more pronounced increase in the Shannon index within the first five years of life, followed by a slightly attenuated rise (*P*=0.005), which suggested a gradual change in respiratory microbiota diversity across the age ranges (**Figure 2B, C**). To assess microbial variation in overall community composition, we first analyzed the Bray-Curtis dissimilarities across four age groups and found children aged < 2 years experienced greater variation compared to other groups (**Figure 2D**). The PCoA results showed a strong separation among four age groups with 24.4% of the variance explained (**Figure 2E**, PERMANOVA *P <*0.001). In adolescents, we also observed a positive association between age and three dominant respiratory microbial clusters characterized by high relative abundance of Firmicutes, Fusobacteriota, and Actinobacteriota. By contrast, the cluster dominated by Bacteroidota and Proteobacteria was higher in younger children (**Figure 2F**). At the genus level, we also identified strong positive associations between the relative abundance of *Prevotella*, *Fusobacterium*, *Haemophilus*, *Rothia*, and age. (**Figure 2G, H**). We further used the linear discriminant analysis effect size method to identify the characteristics of the individual differences in microbiota compositions and found 664 ASVs among four age groups (**Supplementary Table 1**).

### RSV co-infection exacerbates SP-induced LRTI microbiota disorders

**Figure 3:**
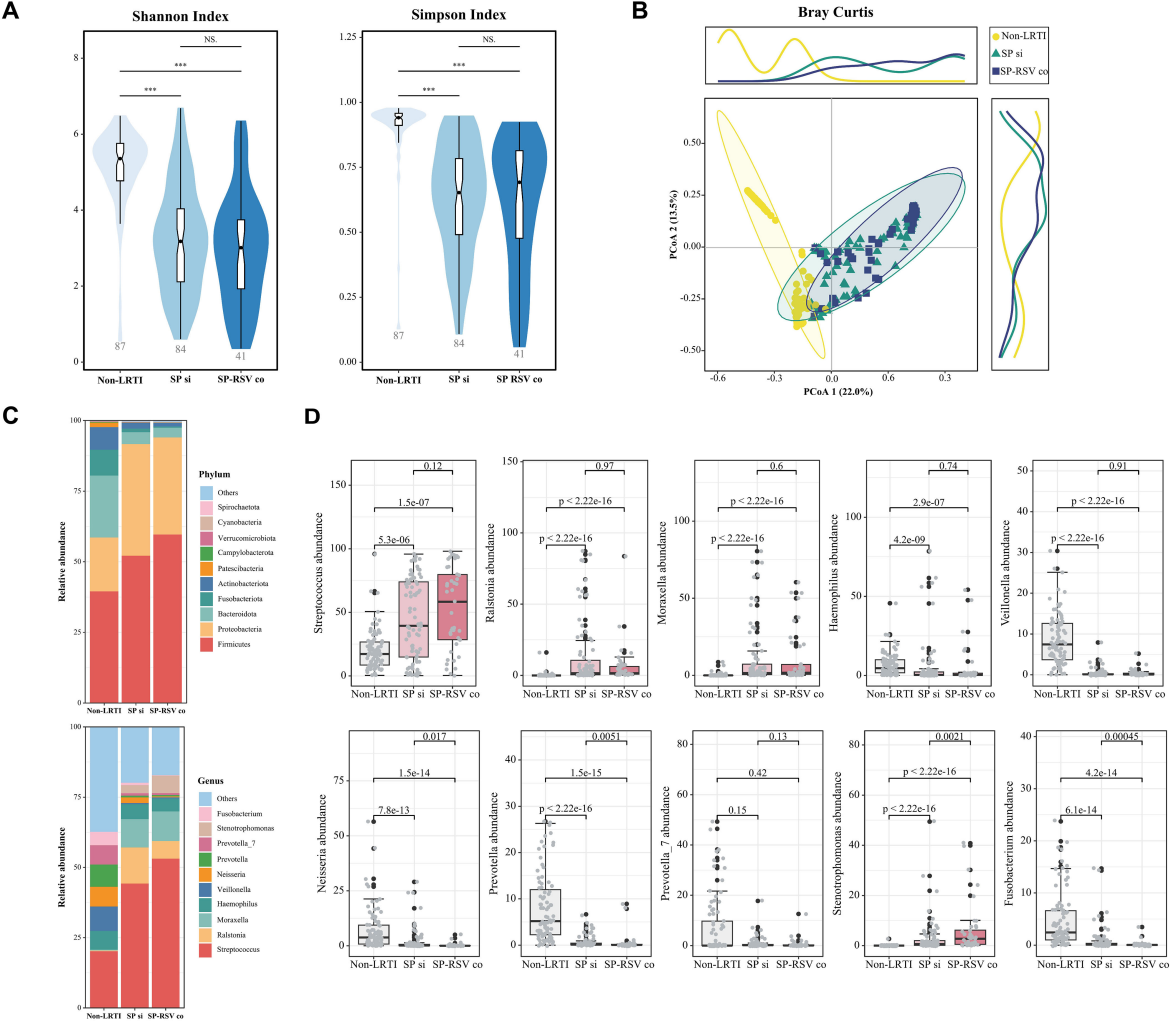
Respiratory infections are correlated to specific lower respiratory tract microbiota disorders. **(A)** Shannon and Simpson diversities among three groups. *P* values were calculated using Wilcoxon rank sum test. **(B)** Principal coordinate analysis (PCoA) based on Bray-Curtis dissimilarities among three groups. **(C)** Relative abundance of phyla and genera among three groups. **(D)** Relative abundance at top 10 genera level among three groups (compared with non-LRTI group). ***p < 0.001; NS p>0.05.

To assess the connection between the pathogen infection and LRT microbiota, we first compared the alpha-diversity and performed PCoA analysis based on Bray-Curtis distance to reveal the microbial differences between the non-LRTI and LRTI children (**Supplementary Figure 1**). The results showed that children with LRTI (either the SP single infection or SP-RSV co-infection group) had lower alpha-diversity and exhibited significant changes in the composition of the respiratory tract microbiota when compared to the non-LRTI children (**Figure 3A, B**). At the phylum level, the most abundant phyla in the two LRTI subgroups were Firmicutes and Proteobacteria. However, the abundance of Bacteroidota, Fusobacteriota, and Actinobacteriota was significantly lower than in non-LRTI controls (**Figure 3C**). A significantly increased abundance of *Streptococcus* was observed in the LRTI subgroups, particularly in the SP-RSV co-infection children (**Figure 3C, D**).

**Figure 4:**
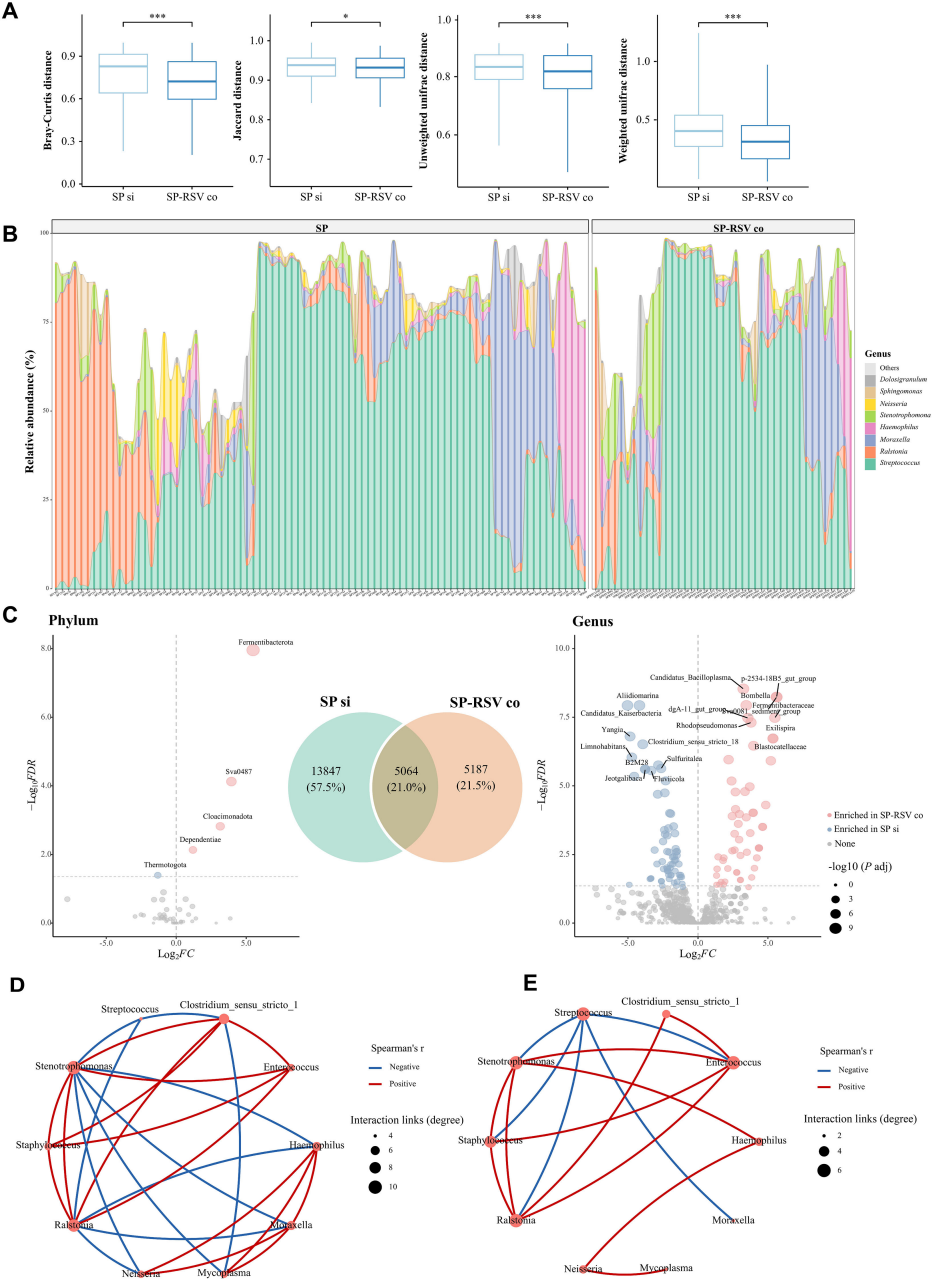
Variations in the composition of lower respiratory tract microbiota in children with *Streptococcus pneumoniae* single infection and *Streptococcus pneumoniae*-respiratory syncytial virus co-infection. **(A)** Four beta-diversity indicates dissimilarities between *Streptococcus pneumoniae* single infection and *Streptococcus pneumoniae*-respiratory syncytial virus co-infection groups. **(B)** Relative abundance of genera in each individual. **(C)** Venn plot showed the number of distinctive and common amplicon sequence variants (ASVs) between two groups. Volcano plots highlight the significant differences in lower respiratory microbiota of children in the *Streptococcus pneumoniae* single infection and *Streptococcus pneumoniae*-respiratory syncytial virus co-infection groups. Metastats was used to compare differences in the relative abundance of species between two groups. Bacteria with a BH-adjusted *P* values <0.05 (adjusted per contrast) and a log2-fold change of 1.0 were deemed significantly different. **(D)** Interaction networks of taxa at genera level in *Streptococcus pneumoniae* single infection children. **(E)** Interaction networks of taxa at genera level in *Streptococcus pneumoniae*-respiratory syncytial virus co-infection children. Only significant (adjusted *P* values <0.05) correlations are displayed. Node size scales with the total number of interaction links per genus (degree centrality). The red lines indicate positive genera interactions, and the blue lines indicate negative interactions. SP si, *Streptococcus pneumoniae* single infection; SP-RSV co, Streptococcus pneumoniae-respiratory syncytial virus co-infection. *P <0.05; ***P <0.001.

The respiratory tract microbiota was then subjected to further analysis between two infection subgroups. Children with SP-RSV co-infections exhibited significantly lower variation in their respiratory tract microbiota than those with SP single infection (all *P <*0.05, **Figure 4A**). At the individual level, despite being diagnosed with SP single infection or SP-RSV co-infection, some of the children exhibited a significant over-proliferation of *Haemophilus*, *Ralstonia*, and *Moraxella* as the dominant microbiota (**Figure 4B**). Specifically, in all LRTI children, Haemophilus abundance exceeded 50% in five patients (4.0%), Ralstonia in nine patients (7.2%), and Moraxella in 11 patients (8.8%). Additionally, we also found that the number of ASVs unique to children with SP-RSV co-infection (21.5%) was lower than that of the SP single infection (57.5%) children. A series of genera were further revealed to contribute to the distinguish of the two infection subgroups using MetaStats analysis (**Figure 4C**). A co-occurrence analysis of the dominant genera was performed to explore the potential respiratory tract microbiota interplay. Intriguingly, we observed that the competitive interactions among respiratory bacteria were more complex in the SP single infection group and simpler in the SP-RSV co-infection group, while *Streptococcus* suppressed the growth of more microbes in the SP-RSV co-infection group (**Figure 4D-E**).

### Severe disease presentations caused by SP-RSV co-infection and the underlying mechanism of microbiota

**Figure 5:**
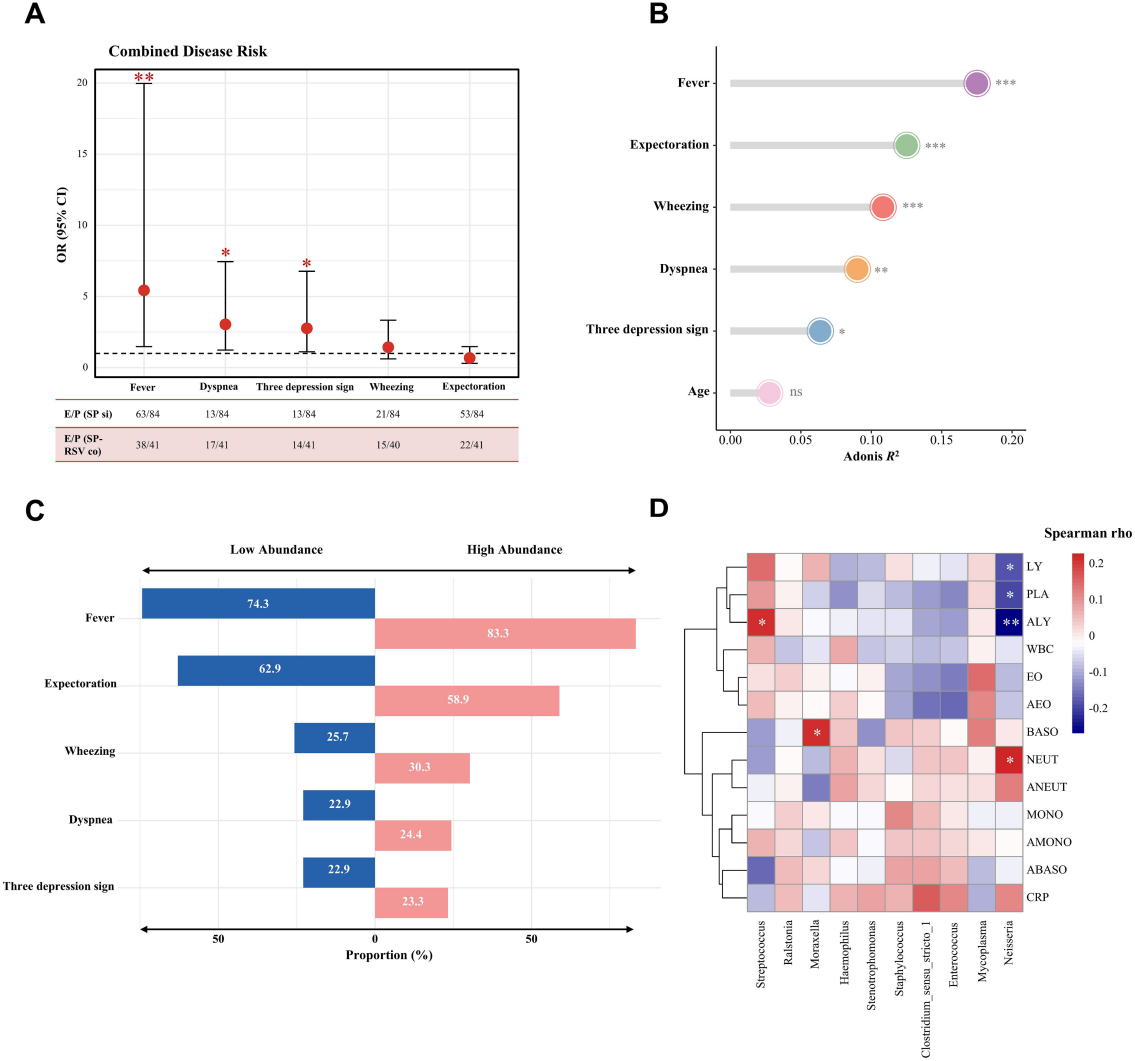
Association of clinical symptoms and lower respiratory tract microbiota in children with *Streptococcus pneumoniae* single infection and *Streptococcus pneumoniae*-respiratory syncytial virus co-infection. **(A)** Forest plot for the risk factors associated with *Streptococcus pneumoniae*-respiratory syncytial virus co-infection when compared with the *Streptococcus pneumoniae* single infection group. The odds ratio (OR) was based on the logistic regression model and adjusted for age. E/P=Events/Patients; SP si=*Streptococcus pneumoniae*; SP-RSV co=*Streptococcus pneumoniae*-respiratory syncytial virus co-infection. **(B)** Variance in the lower respiratory microbiota composition explained by potential factors was assessed through permutational multivariate analysis of variance (PERMANOVA) analysis. The *P* value was determined through 999 permutations. **(C)** The proportion of clinical symptoms in different relative abundance of *Streptococcus pneumoniae*. High abundance was classified as >20% *Streptococcus pneumoniae* reads (based on distribution in **Figure 4C**). **(D)** Correlation between the relative abundance of top 10 lower respiratory bacteria genera and the clinical blood routine indices. WBC, White blood cell count; PLA, Platelets count; NEUT, Neutrophil percentage; LY, Lymphocyte percentage; MONO, Monocyte percentage; EO, Eosinophil percentage; BASO, Basophil percentage; ANEUT, Absolute neutrophil cout; ALY, Absolute lymphocytes count; AMONO, Absolute monocytes count; AEO, Absolute eosinophils count; ABASO, Absolute basophil count; CRP, C-reactive protein. Significance levels are indicated as follows: *P <0.05; **P <0.01; ***P <0.001; ns, *P >*0.05.

The risk of clinical presentations was then compared between children with SP single infection and those with SP-RSV co-infection using a logistic regression model, with age as a covariate. The forest plot showed that co-infection children exhibited a higher risk of fever (OR=5.43, *P*=0.003), dyspnea (OR=3.04, *P*=0.015), and three depression sign (OR=2.76, *P*=0.027) in comparison to children with SP single infection (**Figure 5A**). Additionally, redundancy analysis indicated that fever was most strongly associated with respiratory tract microbiota composition (*P <*0.05, **Figure 5B**). To evaluate whether the abundance of *Streptococcus* is associated with clinical complications, we defined a relative abundance of *Streptococcus* exceeding 20% as high abundance and below 20% as low abundance in LRTI children and compared the proportion of clinical presentations between these two groups. We found that the proportion of clinical complications was higher in the high-abundance group compared to the low-abundance group (**Figure 5C**). In addition, the abundance of *Streptococcus* was positively correlated with the absolute lymphocytes count, suggesting an increased severity of LRTI in children. Conversely, we observed a negative correlation between *Neisseria* (lower abundance in the SP-RSV co-infection group) and absolute lymphocytes count (**Figure 5D**).

### Random forest diagnostic model of SP single infection and SP-RSV co-infection groups

**Figure 6:**
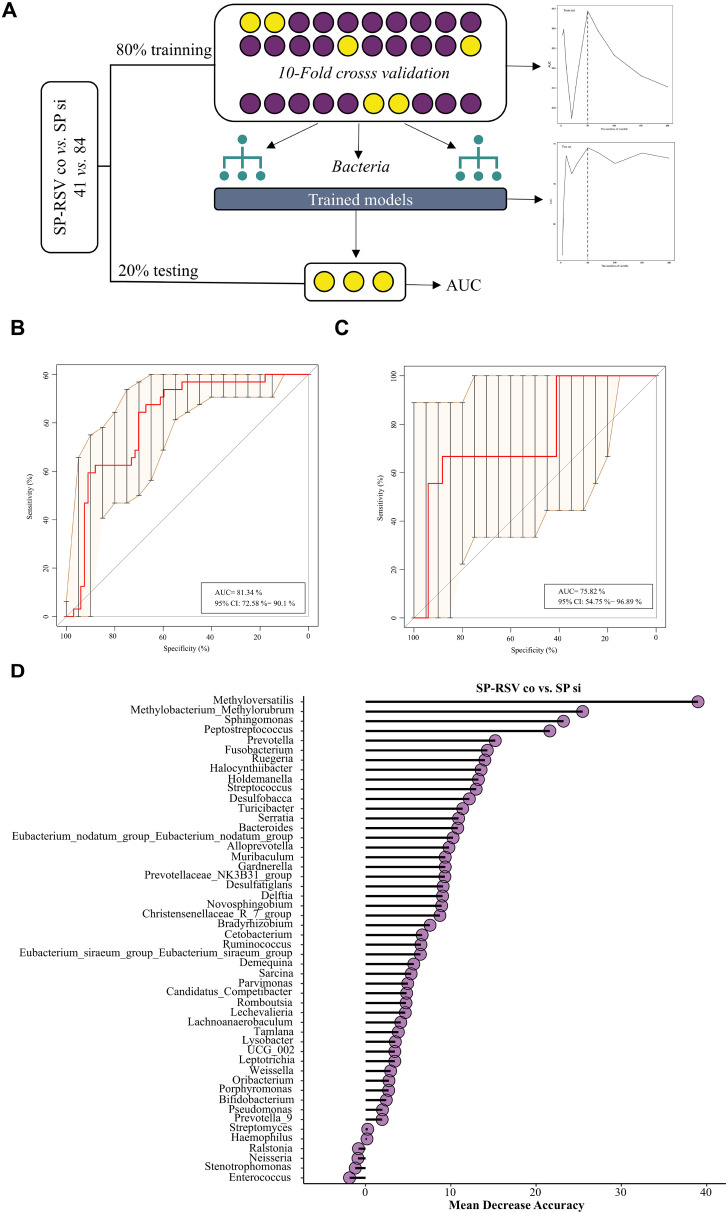
Random forest models for the diagnosis of respiratory *Streptococcus pneumoniae* single infection and *Streptococcus pneumoniae*-respiratory syncytial virus co-infection. **(A)** Schematic diagram of random forest model development. **(B)** The model constructed from the training set was validated in the *Streptococcus pneumoniae* single infection and *Streptococcus pneumoniae*-respiratory syncytial virus co-infection, and the AUC value was calculated. **(C)** The model constructed from the testing set was validated in the *Streptococcus pneumoniae Streptococcus pneumoniae* single infection and *Streptococcus pneumoniae*-respiratory syncytial virus co-infection, and the AUC value was calculated. **(D)** Mean decrease in the accuracy of the 50 respiratory bacteria markers. AUC=Area under of receiver operating characteristic curve. SP si, *Streptococcus pneumoniae* single infection; SP-RSV co, *Streptococcus pneumoniae*-respiratory syncytial virus co-infection.

Distinct microbial landscapes were observed in children with different infection types, which led to the consideration of whether the dynamic characteristics of respiratory microbes could provide a means to clarify infection types. We then employed a random-forest diagnostic model to assess the effectiveness of microbes in distinguishing children with SP-RSV co-infection from those with SP single infection (**Figure 6A**). The area under the operating receiver characteristics curve of the diagnostic classifier constructed by microbial markers extracted from the training set was 0.813 (**Figure 6B**) and 0.758 in the testing set (**Figure 6C**). The selected genera classifiers with significant Mean Decrease Accuracy are shown in **Figure 6D**.

## Discussion

The human respiratory tract consists of complex heterogeneous, and dynamic microbial communities, which are associated with respiratory health outcomes. In this cross-sectional study, it was established that the respiratory tract microbes present across the human lifespan and at different ages are among the key drivers of its diversity and composition. Furthermore, RSV has been observed to facilitate greater colonization of *Streptococcus* and reduce the abundance of *Neisseria* (Besteman et al., 2024; Claassen-Weitz et al., 2025). This may be a significant contributing factor to the higher incidence of LRTI presentations and more severe disease manifestations in children with SP-RSV co-infection compared to those with SP single infection. According to the characteristics of this RSV-related respiratory microbial dysbiosis, we established the microbial classifiers using a machine learning model to differentiate the likelihood of RSV co-infection in children with SP-induced LRTI.

The healthy colonization of the respiratory microbiome in early childhood has been demonstrated to be closely linked to the shaping of respiratory health in later life (de Steenhuijsen Piters et al., 2022). A plethora of studies have documented the patterns of microbial colonization of the upper respiratory tract (e.g., nasopharyngeal and oropharyngeal microbiome) and their dynamic characteristics in children (Bosch et al., 2017; Teo et al., 2018; Odendaal et al., 2024). However, there is a paucity of research investigating longitudinal dynamics and drivers of microbiota colonization in the LRT. Our data suggested that the LRT of younger children exhibited lower diversity and a higher bacterial variation until it stabilized after 5 years of age. This finding is consistent with the development of the nasopharyngeal microbiota (Odendaal et al., 2024) and contrasts with the intestinal microbiota, which stabilizes around 2−5 years of age (Roswall et al., 2021). In general, early-life microbiota is thought to play an important role in establishing tolerogenic immune pathways to prevent unwanted immune responses to self-antigens and innocuous inhaled antigens (Lloyd and Saglani, 2023). In healthy lower airway, we found that the abundance of potentially harmful bacteria like *Haemophilus* spp, *Leptotrichia*, and *Fusobacterium* was strongly associated with age, which may suggest that a higher risk of respiratory issues as children grow, potentially compromising lung function and increasing susceptibility to infection. Another notable finding is that the abundance of SP was low before the age of 2, but increased thereafter and remains relatively stable until 18 years of age. This results suggested that the respiratory microbiome underwent a crucial developmental phase during early childhood (Pattaroni et al., 2018).

Emerging evidence suggests that early respiratory infections may contribute to a lifelong burden of respiratory diseases and potentially increase the risk of other comorbidities (Achten et al., 2022). Although epidemiologic studies have reported an association between RSV and pneumococcal disease in children (Dagan et al., 2023), the mechanism of interaction between these two pathogens remains incompletely clarified. Studies in the upper respiratory tract has indicated that RSV infection is independent of age and associated with the presence of specific nasopharyngeal microbiota, distinguished by enrichment of *Haemophilus* and SP (de Steenhuijsen Piters et al., 2016; Mitsi et al., 2024). The present results supported that RSV may have a substantial role in driving the burden of SP-induced LRTI. In addition, a distinguished microbial landscape was observed between the SP-associated LRTI with or without RSV co-infection using a random-forest model. We observed an increase in *Streptococcus* abundance in the lower respiratory tract of children with RSV infection, accompanied by a decrease in the abundance of *Neisseria*, *Prevotella*, and *Fusobacterium* which has already been described in the healthy respiratory microbiota (Man et al., 2017). This may be related to RSV interference with the host immune response and enhanced adherence of SP to human respiratory tract epithelial cells (Besteman et al., 2024; Shibata et al., 2020). However, our study did not observe an association between RSV infection and higher *Haemophilus* abundance, suggesting that this link may not always exist (Shilts et al., 2021). Intriguingly, we noticed lower bacterial diversity and a reduction in bacterial interactions among children in the SP-RSV co-infection group, resulting in a simpler genus-level co-occurrence network. We hypothesize that this may be related to RSV suppressing the growth of commensal bacteria, leading to an imbalance in the LRT microbiome, and further research is needed to explore the underlying mechanisms of the host immune response. The microbiota analyses in the present study were based on 16S rRNA sequencing, which provides robust community-level insights. Its resolution is restricted to genus-level taxonomy due to conserved regions in the 16S gene. This precludes precise identification of pathogenic species or strains (Langille et al., 2013). Future studies could integrate shotgun metagenomic sequencing or targeted pathogen PCR to resolve these nuances.

Our results supported that viral infections could further perturb the already challenged host-microbial interface, thereby contributing to the manifestation of the disease phenotype and its severity. Previous studies of nasopharyngeal microbiota have shown that children with RSV infection who were simultaneously colonized with a SP-dominated microbiota profile exhibited more severe disease and showed greater overexpression of genes linked to Toll-like receptor and by neutrophil and macrophage activation and signaling (de Steenhuijsen Piters et al., 2016). Similarly, besides a higher risk of LRTI clinical presentations in SP-RSV co-infection children, we also observed that the abundance of *Streptococcus* and *Neisseria* was concordant with specific blood indicators of inflammation, such as lymphocyte, neutrophil, and platelets. A study of neonatal lambs co-infected with SP and RSV found that, despite lower lung bacterial loads, there was an increased pulmonary neutrophil influx, higher myeloperoxidase levels and alveolar thickening, suggesting increased inflammation (Alnajjar et al., 2021). In addition, SP-RSV co-infection of ciliated airway epithelial cells has been shown to enhance the mucosal inflammatory response and reduce ciliary beat frequency, possibly contributing further to pneumonia severity in mice (Smith et al., 2014).

The differentially abundant ASVs identified between the subgroups may serve as potential diagnostic biomarkers. Specifically, the observed rise or decline in relative abundance of these ASVs within each subgroup could be indicative of pathogen-specific ecological interactions or host immune responses triggered by different pathogens. For instance, certain ASVs enriched in the SP-alone group might be associated with SP colonization dynamics, while those elevated in the SP-RSV coinfection subgroup could represent microbial shifts driven by RSV-induced alterations in the respiratory microenvironment or competitive exclusion between pathogens. These patterns are hypothesized to signify unique pathogenic footprints and host-microbe interplay, providing a microbial signature for differentiating monoinfection from coinfection. Further validation of their diagnostic utility and mechanistic exploration of their roles in infection pathogenesis would serve to strengthen their clinical relevance.

The strengths of our study included the systematic characterization of age-specific changes in the LRT microbiota, which suggested an important role for the microbiome in modulating SP-RSV interactions in children. However, there were several limitations. Firstly, the RSV genotype and viral burden have been shown to be significantly associated with respiratory microbiota composition (Ederveen et al., 2018; Lin et al., 2024). Because of the semi-quantitative PCR test used in detecting the virus and bacteria in this study, the precise quantitative load of SP and RSV were recorded in form of positive or negative. The potential impact of viral and bacterial load on the clinical interpretation of our findings cannot be fully excluded and should be addressed in future studies. The serotyping for SP or genotyping for RSV was also not performed, which limits our ability to assess the impact of specific pathogen subtypes on the respiratory microbiota and clinical outcomes. Secondly, we enrolled most of the study population in urban area of Baoding, a relatively small city in China to avoid the environmental or socioeconomic variables. However, some host and environmental characteristics like antibiotics, tobacco, and family members infection status are reported to be related to the airway microbiome (Bogaert et al., 2023; Paulo et al., 2023; Odendaal et al., 2024), it was not thoroughly assessed due to insufficient data. Thirdly, we lack a control group consisting of children infected solely with RSV. The lack of a control group consisting exclusively of RSV subjects hinders the capacity to make direct attributions regarding the observed effects, as it is not possible to distinguish between the effects of RSV alone and those of RSV in conjunction with another pathogen.

Children expose to a multitude of bacteria and viruses through their airways and the relationship between these different pathogens and the respiratory system is critical in establishing the trajectory for respiratory health, which has lifelong implications. Taken together, this study can help us for a better understanding of the characteristics of SP-RSV interaction and provides direction for pathogen diagnosis of LRTI. Future studies need to address the question of how to translate these findings into potential clinical and public health applications and advance research into the implementation of novel interventions aimed at restoring the local microbiome.

## Data Availability

The data that have been released and presented in the study are deposited in the SRA repository, with the accession number PRJNA1268748.
