# Supplementary material for: Lower respiratory tract co-infection of Streptococcus pneumoniae and respiratory syncytial virus shapes microbial landscape and clinical outcomes in children
Source: Front Cell Infect Microbiol. 2025 Jun 9;15:1593053. doi: 10.3389/fcimb.2025.1593053 (PMC12183261; doi:10.3389/fcimb.2025.1593053)
Supplement: Supplementary file 1 [file Table1.docx]

**Supplemantary Material**

**Table S1. The differential ASVs between different age groups in healthy children**

| **Difference genera in LEfSe analysis** | **Enriched groups** | **LDA value** | ***p* value** | ***q* value** | **Significance** |
| --- | --- | --- | --- | --- | --- |
| k__Bacteria\|p__Firmicutes\|c__Bacilli\|o__Lactobacillales\|f__Streptococcaceae\|g__Streptococcus | 2~4 y | 5.0 | 5.596E-14 | 1.458E-11 | *** |
| k__Bacteria\|p__Firmicutes\|c__Bacilli\|o__Lactobacillales\|f__Streptococcaceae\|g__Streptococcus\| | 2~4 y | 5.0 | 6.457E-14 | 1.458E-11 | *** |
| k__Bacteria\| p__Firmicutes\| c__Bacilli\| o__Lactobacillales\| f__Streptococcaceae\| g__Streptococcus | 10~18 y | 5.0 | 2.102E-12 | 4.077E-11 | *** |
| k__Bacteria\|p__Bacteroidota\|c__Bacteroidia\|o__Bacteroidales\|f__Prevotellaceae\|g__Prevotella_7 | 2~4 y | 5.0 | 4.66E-13 | 2.64E-11 | *** |
| k__Bacteria\| p__Firmicutes\| c__Bacilli\| o__Lactobacillales\| f__Streptococcaceae\| g__Streptococcus\| Unclassified | 10~18 y | 4.9 | 6.704E-13 | 3.071E-11 | *** |
| k__Bacteria\|p__Proteobacteria\|c__Gammaproteobacteria\|o__Burkholderiales\|f__Neisseriaceae\|g__Neisseria | <2 y | 4.8 | 3.876E-13 | 2.584E-11 | *** |
| k__Bacteria\|p__Proteobacteria\|c__Gammaproteobacteria\|o__Pseudomonadales\|f__Pseudomonadaceae\|g__Pseudomonas | <2 y | 4.7 | 7.991E-10 | 7.139E-09 | *** |
| k__Bacteria\|p__Proteobacteria\|c__Gammaproteobacteria\|o__Pseudomonadales\|f__Pseudomonadaceae\|g__Pseudomonas\| | <2 y | 4.7 | 7.991E-10 | 7.139E-09 | *** |
| k__Bacteria\|p__Bacteroidota\|c__Bacteroidia\|o__Bacteroidales\|f__Prevotellaceae\|g__Alloprevotella | <2 y | 4.7 | 6.771E-14 | 1.458E-11 | *** |
| k__Bacteria\| p__Bacteroidota\| c__Bacteroidia\| o__Bacteroidales\| f__Prevotellaceae\| g__Prevotella | 10~18 y | 4.7 | 5.546E-12 | 7.855E-11 | *** |
| k__Bacteria\|p__Firmicutes\|c__Negativicutes\|o__Veillonellales-Selenomonadales\|f__Veillonellaceae\|g__Veillonella | 2~4 y | 4.7 | 4.525E-13 | 2.633E-11 | *** |
| k__Bacteria\| p__Firmicutes\| c__Negativicutes\| o__Veillonellales-Selenomonadales\| f__Veillonellaceae\| g__Veillonella | 5~9 y | 4.6 | 2.155E-12 | 4.142E-11 | *** |
| k__Bacteria\| p__Fusobacteriota\| c__Fusobacteriia\| o__Fusobacteriales\| f__Fusobacteriaceae\| g__Fusobacterium | 10~18 y | 4.5 | 3.964E-13 | 2.584E-11 | *** |
| k__Bacteria\| p__Proteobacteria\| c__Gammaproteobacteria\| o__Pasteurellales\| f__Pasteurellaceae\| g__Haemophilus | 5~9 y | 4.5 | 1.217E-12 | 3.55E-11 | *** |
| k__Bacteria\|p__Bacteroidota\|c__Bacteroidia\|o__Bacteroidales\|f__Prevotellaceae\|g__Prevotella_7\|s__Prevotella_histicola | 2~4 y | 4.5 | 6.295E-12 | 8.316E-11 | *** |
| k__Bacteria\| p__Proteobacteria\| c__Gammaproteobacteria\| o__Burkholderiales\| f__Neisseriaceae\| g__Neisseria | 10~18 y | 4.5 | 1.264E-12 | 3.55E-11 | *** |
| k__Bacteria\| p__Firmicutes\| c__Negativicutes\| o__Veillonellales-Selenomonadales\| f__Veillonellaceae\| g__Veillonella\| s__Unclassified | 5~9 y | 4.5 | 3.908E-12 | 6.01E-11 | *** |
| k__Bacteria\| p__Proteobacteria\| c__Gammaproteobacteria\| o__Pasteurellales\| f__Pasteurellaceae\| g__Haemophilus\| s__Unclassified | 5~9 y | 4.5 | 1.149E-12 | 3.55E-11 | *** |
| k__Bacteria\|p__Proteobacteria\|c__Gammaproteobacteria\|o__Burkholderiales\|f__Neisseriaceae\|g__Neisseria\| | <2 y | 4.5 | 3.004E-13 | 2.505E-11 | *** |
| k__Bacteria\| p__Actinobacteriota\| c__Actinobacteria\| o__Micrococcales\| f__Micrococcaceae\| g__Rothia | 5~9 y | 4.5 | 4.773E-12 | 7.039E-11 | *** |
| k__Bacteria\| p__Actinobacteriota\| c__Actinobacteria\| o__Micrococcales\| f__Micrococcaceae\| g__Rothia\| s__Rothia_mucilaginosa | 5~9 y | 4.4 | 4.825E-12 | 7.067E-11 | *** |
| k__Bacteria\|p__Firmicutes\|c__Negativicutes\|o__Veillonellales-Selenomonadales\|f__Veillonellaceae\|g__Veillonella\| | 2~4 y | 4.4 | 1.311E-12 | 3.55E-11 | *** |
| k__Bacteria\|p__Bacteroidota\|c__Bacteroidia\|o__Bacteroidales\|f__Prevotellaceae\|g__Alloprevotella\| | <2 y | 4.4 | 5.028E-12 | 7.315E-11 | *** |
| k__Bacteria\|p__Proteobacteria\|c__Gammaproteobacteria\|o__Enterobacterales\|f__Pasteurellaceae\|g__Haemophilus | 2~4 y | 4.4 | 1.432E-12 | 3.55E-11 | *** |
| k__Bacteria\| p__Fusobacteriota\| c__Fusobacteriia\| o__Fusobacteriales\| f__Fusobacteriaceae\| g__Fusobacterium\| s__Fusobacterium_periodonticum | 10~18 y | 4.4 | 1.054E-12 | 3.55E-11 | *** |
| k__Bacteria\|p__Proteobacteria\|c__Gammaproteobacteria\|o__Enterobacterales\|f__Pasteurellaceae\|g__Haemophilus\| | 2~4 y | 4.4 | 1.301E-12 | 3.55E-11 | *** |
| k__Bacteria\|p__Bacteroidota\|c__Bacteroidia\|o__Bacteroidales\|f__Prevotellaceae\|g__Prevotella_7\|s__Prevotella_melaninogenica | 2~4 y | 4.4 | 1.135E-12 | 3.55E-11 | *** |
| k__Bacteria\|p__Bacteroidota\|c__Bacteroidia\|o__Bacteroidales\|f__Prevotellaceae\|g__Prevotella_7\| | 2~4 y | 4.4 | 4.039E-10 | 3.831E-09 | *** |
| k__Bacteria\|p__Bacteroidota\|c__Bacteroidia\|o__Bacteroidales\|f__Prevotellaceae\|g__Prevotella | 2~4 y | 4.4 | 9.217E-13 | 3.55E-11 | *** |
| k__Bacteria\| p__Fusobacteriota\| c__Fusobacteriia\| o__Fusobacteriales\| f__Leptotrichiaceae\| g__Leptotrichia | 10~18 y | 4.4 | 6.241E-12 | 8.316E-11 | *** |
| k__Bacteria\|p__Firmicutes\|c__Negativicutes\|o__Veillonellales-Selenomonadales\|f__Veillonellaceae\|g__Veillonella\|s__Veillonella_atypica | 2~4 y | 4.3 | 2.236E-12 | 4.261E-11 | *** |
| k__Bacteria\| p__Actinobacteriota\| c__Actinobacteria\| o__Actinomycetales\| f__Actinomycetaceae\| g__Actinomyces | 10~18 y | 4.3 | 1.849E-12 | 3.791E-11 | *** |
| k__Bacteria\| p__Proteobacteria\| c__Gammaproteobacteria\| o__Burkholderiales\| f__Neisseriaceae\| g__Neisseria\| Unclassified | 10~18 y | 4.3 | 2.866E-12 | 4.977E-11 | *** |
| k__Bacteria\|p__Fusobacteriota\|c__Fusobacteriia\|o__Fusobacteriales\|f__Leptotrichiaceae\|g__Leptotrichia | 2~4 y | 4.3 | 3.054E-12 | 5.156E-11 | *** |
| k__Bacteria\| p__Bacteroidota\| c__Bacteroidia\| o__Bacteroidales\| f__Prevotellaceae\| g__Prevotella\| s__Prevotella_melaninogenica | 5~9 y | 4.2 | 3.591E-12 | 5.727E-11 | *** |
| k__Bacteria\|p__Proteobacteria\|c__Gammaproteobacteria\|o__Burkholderiales\|f__Neisseriaceae\|g__Neisseria\|s__Neisseria_perflava | 2~4 y | 4.2 | 4.252E-05 | 0.0002164 | *** |
| k__Bacteria\|p__Actinobacteriota\|c__Actinobacteria\|o__Micrococcales\|f__Micrococcaceae\|g__Rothia | 2~4 y | 4.2 | 2.3E-12 | 4.344E-11 | *** |
| k__Bacteria\| p__Bacteroidota\| c__Bacteroidia\| o__Bacteroidales\| f__Prevotellaceae\| g__Alloprevotella | 10~18 y | 4.1 | 4.177E-12 | 6.378E-11 | *** |
| k__Bacteria\| p__Bacteroidota\| c__Bacteroidia\| o__Bacteroidales\| f__Porphyromonadaceae\| g__Porphyromonas | 10~18 y | 4.1 | 2.935E-12 | 5.015E-11 | *** |
| k__Bacteria\| p__Actinobacteriota\| c__Actinobacteria\| o__Actinomycetales\| f__Actinomycetaceae\| g__Actinomyces\| s__Schaalia_odontolytica | 10~18 y | 4.1 | 2.826E-12 | 4.947E-11 | *** |
| k__Bacteria\| p__Firmicutes\| c__Bacilli\| o__Lactobacillales\| f__Streptococcaceae\| g__Streptococcus\| s__Streptococcus_salivarius | 10~18 y | 4.1 | 2.035E-11 | 2.394E-10 | *** |
| k__Bacteria\| p__Bacteroidota\| c__Bacteroidia\| o__Bacteroidales\| f__Porphyromonadaceae\| g__Porphyromonas\| s__Unclassified | 5~9 y | 4.1 | 3.538E-12 | 5.685E-11 | *** |
| k__Bacteria\|p__Fusobacteriota\|c__Fusobacteriia\|o__Fusobacteriales\|f__Fusobacteriaceae\|g__Fusobacterium | 2~4 y | 4.1 | 3.128E-10 | 2.993E-09 | *** |
| k__Bacteria\| p__Fusobacteriota\| c__Fusobacteriia\| o__Fusobacteriales\| f__Leptotrichiaceae\| g__Leptotrichia\| s__Leptotrichia_sp. | 10~18 y | 4.1 | 1.195E-11 | 1.496E-10 | *** |
| k__Bacteria\| p__Firmicutes\| c__Clostridia\| o__Lachnospirales\| f__Lachnospiraceae\| g__Lachnoanaerobaculum | 10~18 y | 4.1 | 1.375E-12 | 3.55E-11 | *** |
| k__Bacteria\|p__Bacteroidota\|c__Bacteroidia\|o__Bacteroidales\|f__Porphyromonadaceae\|g__Porphyromonas | 2~4 y | 4.1 | 3.231E-12 | 5.239E-11 | *** |
| k__Bacteria\| p__Firmicutes\| c__Clostridia\| o__Lachnospirales\| f__Lachnospiraceae\| g__Lachnoanaerobaculum\| s__Unclassified | 10~18 y | 4.1 | 1.601E-12 | 3.629E-11 | *** |
| k__Bacteria\|p__Proteobacteria\|c__Gammaproteobacteria\|o__Burkholderiales\|f__Burkholderiaceae\|g__Ralstonia | <2 y | 4.0 | 4.606E-07 | 2.96E-06 | *** |
| k__Bacteria\|p__Proteobacteria\|c__Gammaproteobacteria\|o__Burkholderiales\|f__Burkholderiaceae\|g__Ralstonia\| | <2 y | 4.0 | 4.606E-07 | 2.96E-06 | *** |
| k__Bacteria\| p__Firmicutes\| c__Bacilli\| o__Staphylococcales\| f__Gemellaceae\| g__Gemella | 10~18 y | 4.0 | 1.503E-12 | 3.55E-11 | *** |
| k__Bacteria\| p__Firmicutes\| c__Bacilli\| o__Staphylococcales\| f__Gemellaceae\| g__Gemella\| s__Unclassified | 10~18 y | 4.0 | 1.502E-12 | 3.55E-11 | *** |
| k__Bacteria\| p__Bacteroidota\| c__Bacteroidia\| o__Bacteroidales\| f__Prevotellaceae\| g__Alloprevotella\| s__Prevotella_sp. | 5~9 y | 4.0 | 1.359E-12 | 3.55E-11 | *** |
| k__Bacteria\| p__Firmicutes\| c__Bacilli\| o__Erysipelotrichales\| f__Erysipelotrichaceae\| g__Solobacterium | 10~18 y | 4.0 | 1.748E-12 | 3.691E-11 | *** |
| k__Bacteria\| p__Firmicutes\| c__Bacilli\| o__Erysipelotrichales\| f__Erysipelotrichaceae\| g__Solobacterium\| s__Solobacterium_moorei | 10~18 y | 4.0 | 1.748E-12 | 3.691E-11 | *** |
| k__Bacteria\|p__Bacteroidota\|c__Bacteroidia\|o__Bacteroidales\|f__Prevotellaceae\|g__Prevotella\|s__Prevotella_pallens | 2~4 y | 4.0 | 2.613E-11 | 3.041E-10 | *** |
| k__Bacteria\| p__Bacteroidota\| c__Bacteroidia\| o__Bacteroidales\| f__Prevotellaceae\| g__Prevotella\| s__Prevotella_pallens | 10~18 y | 4.0 | 1.308E-11 | 1.619E-10 | *** |
| k__Bacteria\|p__Actinobacteriota\|c__Actinobacteria\|o__Actinomycetales\|f__Actinomycetaceae\|g__Actinomyces | 2~4 y | 3.9 | 8.738E-14 | 1.543E-11 | *** |
| k__Bacteria\|p__Proteobacteria\|c__Gammaproteobacteria\|o__Burkholderiales\|f__Rhodocyclaceae\|g__Methyloversatilis | <2 y | 3.9 | 2.635E-08 | 1.848E-07 | *** |
| k__Bacteria\|p__Proteobacteria\|c__Gammaproteobacteria\|o__Burkholderiales\|f__Rhodocyclaceae\|g__Methyloversatilis\| | <2 y | 3.9 | 2.635E-08 | 1.848E-07 | *** |
| k__Bacteria\|p__Fusobacteriota\|c__Fusobacteriia\|o__Fusobacteriales\|f__Fusobacteriaceae\|g__Fusobacterium\|s__Fusobacterium_periodonticum | 2~4 y | 3.9 | 4.123E-07 | 2.69E-06 | *** |
| k__Bacteria\|p__Firmicutes\|c__Bacilli\|o__Lactobacillales\|f__Lactobacillaceae\|g__Lactobacillus | <2 y | 3.9 | 5.005E-05 | 0.0002512 | *** |
| k__Bacteria\|p__Bacteroidota\|c__Bacteroidia\|o__Bacteroidales\|f__Porphyromonadaceae\|g__Porphyromonas\|s__Porphyromonas_sp_oral_clone_HF001 | <2 y | 3.9 | 1.057E-09 | 9.212E-09 | *** |
| k__Bacteria\| p__Firmicutes\| c__Clostridia\| o__Peptostreptococcales-Tissierellales\| f__Peptostreptococcaceae\| g__Peptostreptococcus | 10~18 y | 3.9 | 9.362E-13 | 3.55E-11 | *** |
| k__Bacteria\| p__Firmicutes\| c__Clostridia\| o__Peptostreptococcales-Tissierellales\| f__Peptostreptococcaceae\| g__Peptostreptococcus\| s__Unclassified | 10~18 y | 3.9 | 1.006E-12 | 3.55E-11 | *** |
| k__Bacteria\|p__Actinobacteriota\|c__Actinobacteria\|o__Micrococcales\|f__Micrococcaceae\|g__Rothia\| | 2~4 y | 3.9 | 6.33E-12 | 8.316E-11 | *** |
| k__Bacteria\| p__Fusobacteriota\| c__Fusobacteriia\| o__Fusobacteriales\| f__Leptotrichiaceae\| g__Leptotrichia\| s__Unclassified | 10~18 y | 3.9 | 2.062E-12 | 4.077E-11 | *** |
| k__Bacteria\| p__Proteobacteria\| c__Gammaproteobacteria\| o__Burkholderiales\| f__Neisseriaceae\| g__Neisseria\| s__Unclassified | 10~18 y | 3.9 | 8.312E-13 | 3.55E-11 | *** |
| k__Bacteria\|p__Bacteroidota\|c__Bacteroidia\|o__Bacteroidales\|f__Prevotellaceae\|g__Prevotella\|s__Prevotella_salivae | 2~4 y | 3.9 | 8.25E-11 | 8.925E-10 | *** |
| k__Bacteria\| p__Bacteroidota\| c__Bacteroidia\| o__Bacteroidales\| f__Prevotellaceae\| g__Prevotella\| Unclassified | 10~18 y | 3.9 | 5.759E-11 | 6.359E-10 | *** |
| k__Bacteria\|p__Fusobacteriota\|c__Fusobacteriia\|o__Fusobacteriales\|f__Leptotrichiaceae\|g__Leptotrichia\| | 2~4 y | 3.9 | 1.491E-10 | 1.514E-09 | *** |
| k__Bacteria\| p__Patescibacteria\| c__Saccharimonadia\| o__Saccharimonadales\| f__Saccharimonadaceae\| g__TM7x | 10~18 y | 3.8 | 2.382E-12 | 4.346E-11 | *** |
| k__Bacteria\| p__Patescibacteria\| c__Saccharimonadia\| o__Saccharimonadales\| f__Saccharimonadaceae\| g__TM7x\| s__Unclassified | 10~18 y | 3.8 | 2.381E-12 | 4.346E-11 | *** |
| k__Bacteria\| p__Fusobacteriota\| c__Fusobacteriia\| o__Fusobacteriales\| f__Fusobacteriaceae\| g__Fusobacterium\| s__Fusobacterium_nucleatum | 10~18 y | 3.8 | 2.588E-12 | 4.605E-11 | *** |
| k__Bacteria\| p__Firmicutes\| c__Bacilli\| o__Lactobacillales\| f__P5D1-392\| g__Unclassified | 5~9 y | 3.8 | 1.836E-11 | 2.185E-10 | *** |
| k__Bacteria\| p__Firmicutes\| c__Bacilli\| o__Lactobacillales\| f__P5D1-392\| g__Unclassified\| s__Unclassified | 5~9 y | 3.8 | 1.837E-11 | 2.185E-10 | *** |
| k__Bacteria\|p__Fusobacteriota\|c__Fusobacteriia\|o__Fusobacteriales\|f__Leptotrichiaceae\|g__Leptotrichia\|s__Leptotrichia_sp_oral_clone_EI013 | <2 y | 3.8 | 2.342E-05 | 0.0001209 | *** |
| k__Bacteria\| p__Firmicutes\| c__Clostridia\| o__Lachnospirales\| f__Lachnospiraceae\| g__Oribacterium | 10~18 y | 3.8 | 3.237E-12 | 5.239E-11 | *** |
| k__Bacteria\|p__Verrucomicrobiota\|c__Verrucomicrobiae\|o__Verrucomicrobiales\|f__Akkermansiaceae\|g__Akkermansia | <2 y | 3.8 | 1.431E-06 | 8.704E-06 | *** |
| k__Bacteria\|p__Fusobacteriota\|c__Fusobacteriia\|o__Fusobacteriales\|f__Leptotrichiaceae\|g__Streptobacillus | <2 y | 3.8 | 9.149E-07 | 5.693E-06 | *** |
| k__Bacteria\|p__Verrucomicrobiota\|c__Verrucomicrobiae\|o__Verrucomicrobiales\|f__Akkermansiaceae\|g__Akkermansia\| | <2 y | 3.8 | 1.431E-06 | 8.704E-06 | *** |
| k__Bacteria\|p__Patescibacteria\|c__Saccharimonadia\|o__Saccharimonadales\|f__Saccharimonadaceae\|g__Candidatus_Saccharimonas | <2 y | 3.8 | 2.163E-05 | 0.000113 | *** |
| k__Bacteria\|p__Actinobacteriota\|c__Actinobacteria\|o__Micrococcales\|f__Micrococcaceae\|g__Rothia\|s__Rothia_mucilaginosa | <2 y | 3.8 | 0.0002329 | 0.0010855 | ** |
| k__Bacteria\|p__Firmicutes\|c__Negativicutes\|o__Veillonellales-Selenomonadales\|f__Veillonellaceae\|g__Veillonella\|s__Veillonella_sp_oral_taxon_780 | <2 y | 3.8 | 5.269E-09 | 4.066E-08 | *** |
| k__Bacteria\|p__Firmicutes\|c__Bacilli\|o__Staphylococcales\|f__Staphylococcaceae\|g__Staphylococcus | <2 y | 3.8 | 1.231E-05 | 6.61E-05 | *** |
| k__Bacteria\|p__Firmicutes\|c__Bacilli\|o__Staphylococcales\|f__Staphylococcaceae\|g__Staphylococcus\| | <2 y | 3.8 | 1.231E-05 | 6.61E-05 | *** |
| k__Bacteria\| p__Bacteroidota\| c__Bacteroidia\| o__Bacteroidales\| f__Prevotellaceae\| g__Prevotella\| s__Prevotella_histicola | 5~9 y | 3.7 | 1.623E-09 | 1.354E-08 | *** |
| k__Bacteria\| p__Proteobacteria\| c__Gammaproteobacteria\| o__Burkholderiales\| f__Neisseriaceae\| g__Neisseria\| s__Neisseria_subflava | 10~18 y | 3.7 | 3.778E-09 | 2.99E-08 | *** |
| k__Bacteria\|p__Bacteroidota\|c__Bacteroidia\|o__Bacteroidales\|f__Porphyromonadaceae\|g__Porphyromonas\| | <2 y | 3.7 | 2.953E-10 | 2.851E-09 | *** |
| k__Bacteria\|p__Firmicutes\|c__Bacilli\|o__Lactobacillales\|f__Lactobacillaceae\|g__Lactobacillus\| | <2 y | 3.7 | 5.005E-05 | 0.0002512 | *** |
| k__Bacteria\|p__Actinobacteriota\|c__Actinobacteria\|o__Actinomycetales\|f__Actinomycetaceae\|g__Actinomyces\|s__Schaalia_odontolytica | 2~4 y | 3.7 | 4.408E-13 | 2.633E-11 | *** |
| k__Bacteria\| p__Proteobacteria\| c__Gammaproteobacteria\| o__Burkholderiales\| f__Oxalobacteraceae\| g__Massilia | 10~18 y | 3.7 | 1.22E-06 | 7.527E-06 | *** |
| k__Bacteria\|p__Firmicutes\|c__Bacilli\|o__Staphylococcales\|f__Gemellaceae\|g__Gemella | 2~4 y | 3.7 | 2.083E-12 | 4.077E-11 | *** |
| k__Bacteria\|p__Patescibacteria\|c__Gracilibacteria\|o__Absconditabacteriales_(SR1)\|f__unidentified_Absconditabacteriales_(SR1)\|g__unidentified_Absconditabacteriales_(SR1) | <2 y | 3.7 | 0.0005267 | 0.0023142 | ** |
| k__Bacteria\|p__Bacteroidota\|c__Bacteroidia\|o__Flavobacteriales\|f__Weeksellaceae\|g__Bergeyella | 2~4 y | 3.7 | 1.73E-09 | 1.429E-08 | *** |
| k__Bacteria\|p__Bacteroidota\|c__Bacteroidia\|o__Flavobacteriales\|f__Weeksellaceae\|g__Chryseobacterium | <2 y | 3.7 | 4.398E-07 | 2.844E-06 | *** |
| k__Bacteria\|p__Bacteroidota\|c__Bacteroidia\|o__Flavobacteriales\|f__Weeksellaceae\|g__Chryseobacterium\| | <2 y | 3.7 | 4.398E-07 | 2.844E-06 | *** |
| k__Bacteria\| p__Firmicutes\| c__Negativicutes\| o__Veillonellales-Selenomonadales\| f__Veillonellaceae\| g__Veillonella\| Unclassified | 10~18 y | 3.6 | 1.945E-13 | 2.43E-11 | *** |
| k__Bacteria\| p__Actinobacteriota\| c__Coriobacteriia\| o__Coriobacteriales\| f__Atopobiaceae\| g__Atopobium | 10~18 y | 3.6 | 6.563E-12 | 8.411E-11 | *** |
| k__Bacteria\| p__Proteobacteria\| c__Gammaproteobacteria\| o__Burkholderiales\| f__Oxalobacteraceae\| g__Massilia\| s__Massilia_timonae | 10~18 y | 3.6 | 6.34E-06 | 3.536E-05 | *** |
| k__Bacteria\| p__Actinobacteriota\| c__Coriobacteriia\| o__Coriobacteriales\| f__Atopobiaceae\| g__Atopobium\| s__Unclassified | 10~18 y | 3.6 | 6.155E-11 | 6.761E-10 | *** |
| k__Bacteria\| p__Firmicutes\| c__Bacilli\| o__Lactobacillales\| f__Streptococcaceae\| g__Streptococcus\| s__Streptococcus_parasanguinis | 5~9 y | 3.6 | 6.988E-11 | 7.598E-10 | *** |
| k__Bacteria\|p__Fusobacteriota\|c__Fusobacteriia\|o__Fusobacteriales\|f__Fusobacteriaceae\|g__Fusobacterium\| | 2~4 y | 3.6 | 9.516E-09 | 7.089E-08 | *** |
| k__Bacteria\| p__Bacteroidota\| c__Bacteroidia\| o__Bacteroidales\| f__Prevotellaceae\| g__Prevotella\| s__Prevotella_sp. | 5~9 y | 3.5 | 1.327E-11 | 1.633E-10 | *** |
| k__Bacteria\| p__Proteobacteria\| c__Gammaproteobacteria\| o__Pasteurellales\| f__Pasteurellaceae\| g__Haemophilus\| s__Haemophilus_haemolyticus | 10~18 y | 3.5 | 7.935E-07 | 4.966E-06 | *** |
| k__Bacteria\|p__Campylobacterota\|c__Campylobacteria\|o__Campylobacterales\|f__Campylobacteraceae\|g__Campylobacter | 2~4 y | 3.5 | 6.145E-13 | 2.904E-11 | *** |
| k__Bacteria\| p__Firmicutes\| c__Negativicutes\| o__Veillonellales-Selenomonadales\| f__Veillonellaceae\| g__Megasphaera | 10~18 y | 3.5 | 4.922E-11 | 5.577E-10 | *** |
| k__Bacteria\| p__Firmicutes\| c__Negativicutes\| o__Veillonellales-Selenomonadales\| f__Veillonellaceae\| g__Megasphaera\| s__Megasphaera_micronuciformis | 10~18 y | 3.5 | 5.006E-11 | 5.643E-10 | *** |
| k__Bacteria\|p__Campylobacterota\|c__Campylobacteria\|o__Campylobacterales\|f__Campylobacteraceae\|g__Campylobacter\|s__Campylobacter_concisus | 2~4 y | 3.5 | 1.523E-12 | 3.55E-11 | *** |
| k__Bacteria\| p__Bacteroidota\| c__Bacteroidia\| o__Bacteroidales\| f__Prevotellaceae\| g__Prevotella\| s__Prevotella_salivae | 5~9 y | 3.5 | 6.492E-10 | 5.948E-09 | *** |
| k__Bacteria\| p__Firmicutes\| c__Clostridia\| o__Lachnospirales\| f__Lachnospiraceae\| g__Stomatobaculum | 10~18 y | 3.5 | 5.778E-12 | 7.979E-11 | *** |
| k__Bacteria\| p__Firmicutes\| c__Clostridia\| o__Lachnospirales\| f__Lachnospiraceae\| g__Stomatobaculum\| s__Unclassified | 10~18 y | 3.5 | 5.78E-12 | 7.979E-11 | *** |
| k__Bacteria\|p__Bacteroidota\|c__Bacteroidia\|o__Bacteroidales\|f__Prevotellaceae\|g__Prevotella_7\|s__Prevotella_jejuni | 2~4 y | 3.5 | 1.408E-07 | 9.386E-07 | *** |
| k__Bacteria\|p__Proteobacteria\|c__Gammaproteobacteria\|o__Pseudomonadales\|f__Moraxellaceae\|g__Moraxella | <2 y | 3.4 | 0.0049497 | 0.0178806 | * |
| k__Bacteria\| p__Patescibacteria\| c__Gracilibacteria\| o__Absconditabacteriales_(SR1)\| f__Unclassified\| g__Unclassified | 10~18 y | 3.4 | 7.486E-09 | 5.675E-08 | *** |
| k__Bacteria\| p__Patescibacteria\| c__Gracilibacteria\| o__Absconditabacteriales_(SR1)\| f__Unclassified\| g__Unclassified\| s__SR1_bacterium | 10~18 y | 3.4 | 7.481E-09 | 5.675E-08 | *** |
| k__Bacteria\|p__Proteobacteria\|c__Gammaproteobacteria\|o__Pseudomonadales\|f__Moraxellaceae\|g__Moraxella\| | <2 y | 3.4 | 0.010676 | 0.0348264 | * |
| k__Bacteria\| p__Bacteroidota\| c__Bacteroidia\| o__Bacteroidales\| f__Prevotellaceae\| g__Prevotella\| s__Prevotella_jejuni | 10~18 y | 3.4 | 4.354E-08 | 3.005E-07 | *** |
| k__Bacteria\| p__Proteobacteria\| c__Gammaproteobacteria\| o__Pasteurellales\| f__Pasteurellaceae\| g__Aggregatibacter | 5~9 y | 3.4 | 9.448E-12 | 1.197E-10 | *** |
| k__Bacteria\| p__Firmicutes\| c__Clostridia\| o__Peptostreptococcales-Tissierellales\| f__Anaerovoracaceae\| g__[Eubacterium]_nodatum_group | 10~18 y | 3.4 | 3.778E-12 | 5.894E-11 | *** |
| k__Bacteria\| p__Firmicutes\| c__Clostridia\| o__Lachnospirales\| f__Lachnospiraceae\| g__Oribacterium\| Unclassified | 5~9 y | 3.4 | 5.634E-12 | 7.928E-11 | *** |
| k__Bacteria\| p__Proteobacteria\| c__Gammaproteobacteria\| o__Pasteurellales\| f__Pasteurellaceae\| g__Aggregatibacter\| s__Unclassified | 5~9 y | 3.4 | 9.888E-12 | 1.245E-10 | *** |
| k__Bacteria\| p__Firmicutes\| c__Clostridia\| o__Peptostreptococcales-Tissierellales\| f__Anaerovoracaceae\| g__[Eubacterium]_nodatum_group\| s__Eubacterium_sulci | 10~18 y | 3.4 | 1.308E-11 | 1.619E-10 | *** |
| k__Bacteria\| p__Firmicutes\| c__Negativicutes\| o__Veillonellales-Selenomonadales\| f__Veillonellaceae\| g__Veillonella\| s__Veillonella_rogosae | 10~18 y | 3.4 | 1.677E-10 | 1.671E-09 | *** |
| k__Bacteria\|p__Firmicutes\|c__Clostridia\|o__Lachnospirales\|f__Lachnospiraceae\|g__Lachnoanaerobaculum | 2~4 y | 3.4 | 1.389E-10 | 1.417E-09 | *** |
| k__Bacteria\| p__Firmicutes\| c__Clostridia\| o__Lachnospirales\| f__Lachnospiraceae\| g__Oribacterium\| s__Unclassified | 10~18 y | 3.4 | 9.814E-10 | 8.625E-09 | *** |
| k__Bacteria\|p__Patescibacteria\|c__Saccharimonadia\|o__Saccharimonadales\|f__Saccharimonadaceae\|g__TM7x | 2~4 y | 3.4 | 4.194E-07 | 2.728E-06 | *** |
| k__Bacteria\| p__Bacteroidota\| c__Bacteroidia\| o__Flavobacteriales\| f__Weeksellaceae\| g__Bergeyella | 10~18 y | 3.4 | 1.918E-10 | 1.894E-09 | *** |
| k__Bacteria\| p__Bacteroidota\| c__Bacteroidia\| o__Flavobacteriales\| f__Weeksellaceae\| g__Bergeyella\| s__Unclassified | 10~18 y | 3.4 | 4.854E-10 | 4.544E-09 | *** |
| k__Bacteria\|p__Actinobacteriota\|c__Coriobacteriia\|o__Coriobacteriales\|f__Atopobiaceae\|g__Atopobium | 2~4 y | 3.3 | 6.633E-12 | 8.451E-11 | *** |
| k__Bacteria\|p__Actinobacteriota\|c__Actinobacteria\|o__Actinomycetales\|f__Actinomycetaceae\|g__Actinomyces\|s__Actinomyces_graevenitzii | 2~4 y | 3.3 | 6.077E-09 | 4.673E-08 | *** |
| k__Bacteria\| p__Proteobacteria\| c__Gammaproteobacteria\| o__Pasteurellales\| f__Pasteurellaceae\| g__Haemophilus\| s__Haemophilus_influenzae | 5~9 y | 3.3 | 3.121E-08 | 2.168E-07 | *** |
| k__Bacteria\| p__Bacteroidota\| c__Bacteroidia\| o__Bacteroidales\| f__Prevotellaceae\| g__Alloprevotella\| s__Alloprevotella_tannerae | 10~18 y | 3.3 | 4.907E-11 | 5.577E-10 | *** |
| k__Bacteria\|p__Proteobacteria\|c__Gammaproteobacteria\|o__Enterobacterales\|f__Yersiniaceae\|g__Serratia | <2 y | 3.3 | 0.0003714 | 0.0016834 | ** |
| k__Bacteria\|p__Proteobacteria\|c__Gammaproteobacteria\|o__Enterobacterales\|f__Yersiniaceae\|g__Serratia\| | <2 y | 3.3 | 0.0003714 | 0.0016834 | ** |
| k__Bacteria\|p__Firmicutes\|c__Bacilli\|o__Lactobacillales\|f__Streptococcaceae\|g__Streptococcus\|s__Streptococcus_peroris | <2 y | 3.3 | 3.628E-06 | 2.083E-05 | *** |
| k__Bacteria\| p__Actinobacteriota\| c__Actinobacteria\| o__Actinomycetales\| f__Actinomycetaceae\| g__Actinomyces\| s__Actinomyces_lingnae | 10~18 y | 3.3 | 1.125E-09 | 9.687E-09 | *** |
| k__Bacteria\|p__Firmicutes\|c__Clostridia\|o__Lachnospirales\|f__Lachnospiraceae\|g__Lachnospiraceae_NK4A136_group | <2 y | 3.3 | 3.674E-07 | 2.411E-06 | *** |
| k__Bacteria\|p__Proteobacteria\|c__Gammaproteobacteria\|o__Burkholderiales\|f__Neisseriaceae\|g__Neisseria\|s__Neisseria_elongata | <2 y | 3.3 | 0.0050843 | 0.0182447 | * |
| k__Bacteria\| p__Firmicutes\| c__Bacilli\| o__Lactobacillales\| f__Streptococcaceae\| g__Streptococcus\| s__Streptococcus_cristatus | 10~18 y | 3.2 | 4.326E-13 | 2.633E-11 | *** |
| k__Bacteria\| p__Firmicutes\| c__Clostridia\| o__Lachnospirales\| f__Lachnospiraceae\| g__Oribacterium\| s__Oribacterium_parvum | 10~18 y | 3.2 | 3.009E-10 | 2.892E-09 | *** |
| k__Bacteria\| p__Fusobacteriota\| c__Fusobacteriia\| o__Fusobacteriales\| f__Leptotrichiaceae\| g__Leptotrichia\| s__Leptotrichia_hofstadii | 5~9 y | 3.2 | 2.575E-05 | 0.0001326 | *** |
| k__Bacteria\|p__Firmicutes\|c__Clostridia\|o__Lachnospirales\|f__Lachnospiraceae\|g__Lachnospiraceae_NK4A136_group\| | <2 y | 3.2 | 0.0046258 | 0.0168801 | * |
| k__Bacteria\|p__Firmicutes\|c__Clostridia\|o__Oscillospirales\|f__Ruminococcaceae\|g__Ruminococcus | <2 y | 3.2 | 0.0014022 | 0.0058055 | ** |
| k__Bacteria\| p__Firmicutes\| c__Clostridia\| o__Peptostreptococcales-Tissierellales\| f__Unclassified\| g__Parvimonas | 10~18 y | 3.2 | 2.651E-13 | 2.505E-11 | *** |
| k__Bacteria\|p__Fusobacteriota\|c__Fusobacteriia\|o__Fusobacteriales\|f__Leptotrichiaceae\|g__Leptotrichia\|s__Leptotrichia_sp_oral_clone_FP036 | 2~4 y | 3.2 | 1.97E-10 | 1.919E-09 | *** |
| k__Bacteria\| p__Bacteroidota\| c__Bacteroidia\| o__Flavobacteriales\| f__Flavobacteriaceae\| g__Capnocytophaga | 10~18 y | 3.2 | 5.593E-11 | 6.207E-10 | *** |
| k__Bacteria\|p__Bacteroidota\|c__Bacteroidia\|o__Bacteroidales\|f__Porphyromonadaceae\|g__Porphyromonas\|s__Porphyromonas_pasteri | 2~4 y | 3.2 | 9.175E-05 | 0.0004552 | *** |
| k__Bacteria\| p__Proteobacteria\| c__Gammaproteobacteria\| o__Pasteurellales\| f__Pasteurellaceae\| g__Actinobacillus | 5~9 y | 3.2 | 4.327E-11 | 4.956E-10 | *** |
| k__Bacteria\| p__Proteobacteria\| c__Gammaproteobacteria\| o__Pasteurellales\| f__Pasteurellaceae\| g__Actinobacillus\| s__Haemophilus_parahaemolyticus | 5~9 y | 3.2 | 1.422E-09 | 1.201E-08 | *** |
| k__Bacteria\| p__Firmicutes\| c__Negativicutes\| o__Veillonellales-Selenomonadales\| f__Selenomonadaceae\| g__Selenomonas | 10~18 y | 3.2 | 2.086E-09 | 1.714E-08 | *** |
| k__Bacteria\|p__Firmicutes\|c__Clostridia\|o__Lachnospirales\|f__Lachnospiraceae\|g__Oribacterium | 2~4 y | 3.2 | 9.06E-11 | 9.753E-10 | *** |
| k__Bacteria\| p__Bacteroidota\| c__Bacteroidia\| o__Bacteroidales\| f__Prevotellaceae\| g__Prevotella\| s__Prevotella_nigrescens | 10~18 y | 3.2 | 1.36E-06 | 8.368E-06 | *** |
| k__Bacteria\| p__Actinobacteriota\| c__Actinobacteria\| o__Corynebacteriales\| f__Corynebacteriaceae\| g__Corynebacterium | 10~18 y | 3.2 | 1.404E-09 | 1.19E-08 | *** |
| k__Bacteria\| p__Firmicutes\| c__Bacilli\| o__Lactobacillales\| f__Carnobacteriaceae\| g__Granulicatella | 10~18 y | 3.2 | 5.141E-12 | 7.384E-11 | *** |
| k__Bacteria\|p__Actinobacteriota\|c__Actinobacteria\|o__Actinomycetales\|f__Actinomycetaceae\|g__Actinomyces\| | 2~4 y | 3.2 | 6.175E-12 | 8.309E-11 | *** |
| k__Bacteria\| p__Firmicutes\| c__Bacilli\| o__Lactobacillales\| f__Carnobacteriaceae\| g__Granulicatella\| s__Unclassified | 10~18 y | 3.2 | 1.545E-11 | 1.869E-10 | *** |
| k__Bacteria\|p__Firmicutes\|c__Bacilli\|o__Lactobacillales\|f__Aerococcaceae\|g__Abiotrophia | 2~4 y | 3.1 | 5.697E-10 | 5.264E-09 | *** |
| k__Bacteria\|p__Actinobacteriota\|c__Coriobacteriia\|o__Coriobacteriales\|f__Atopobiaceae\|g__Atopobium\| | 2~4 y | 3.1 | 5.827E-08 | 3.983E-07 | *** |
| k__Bacteria\| p__Firmicutes\| c__Bacilli\| o__Lactobacillales\| f__Streptococcaceae\| g__Streptococcus\| s__Streptococcus_sanguinis | 10~18 y | 3.1 | 3.505E-11 | 4.057E-10 | *** |
| k__Bacteria\|p__Actinobacteriota\|c__Actinobacteria\|o__Corynebacteriales\|f__Corynebacteriaceae\|g__Corynebacterium | <2 y | 3.1 | 3.151E-09 | 2.531E-08 | *** |
| k__Bacteria\| p__Firmicutes\| c__Clostridia\| o__Peptostreptococcales-Tissierellales\| f__Unclassified\| g__Parvimonas\| s__Unclassified | 10~18 y | 3.1 | 6.972E-10 | 6.334E-09 | *** |
| k__Bacteria\|p__Firmicutes\|c__Clostridia\|o__Oscillospirales\|f__Ruminococcaceae\|g__Ruminococcus\| | <2 y | 3.1 | 0.0014022 | 0.0058055 | ** |
| k__Bacteria\| p__Actinobacteriota\| c__Actinobacteria\| o__Actinomycetales\| f__Actinomycetaceae\| g__Actinomyces\| s__Actinomyces_pacaensis | 5~9 y | 3.1 | 5.538E-07 | 3.517E-06 | *** |
| k__Bacteria\| p__Bacteroidota\| c__Bacteroidia\| o__Bacteroidales\| f__Prevotellaceae\| g__Prevotella\| s__Prevotella_veroralis | 10~18 y | 3.1 | 2.364E-06 | 1.376E-05 | *** |
| k__Bacteria\| p__Firmicutes\| c__Negativicutes\| o__Veillonellales-Selenomonadales\| f__Veillonellaceae\| g__Veillonella\| s__Veillonella_parvula | 10~18 y | 3.1 | 4.42E-12 | 6.702E-11 | *** |
| k__Bacteria\|p__Proteobacteria\|c__Gammaproteobacteria\|o__Enterobacterales\|f__Vibrionaceae\|g__Vibrio | <2 y | 3.1 | 0.0071412 | 0.0241747 | * |
| k__Bacteria\|p__Proteobacteria\|c__Gammaproteobacteria\|o__Enterobacterales\|f__Vibrionaceae\|g__Vibrio\| | <2 y | 3.1 | 0.0071412 | 0.0241747 | * |
| k__Bacteria\| p__Patescibacteria\| c__Saccharimonadia\| o__Saccharimonadales\| f__Unclassified\| g__Unclassified | 10~18 y | 3.1 | 1.949E-10 | 1.908E-09 | *** |
| k__Bacteria\|p__Bacteroidota\|c__Bacteroidia\|o__Flavobacteriales\|f__Flavobacteriaceae\|g__Capnocytophaga | 2~4 y | 3.1 | 1.223E-10 | 1.266E-09 | *** |
| k__Bacteria\|p__Proteobacteria\|c__Gammaproteobacteria\|o__Enterobacterales\|f__Pasteurellaceae\|g__Aggregatibacter | 2~4 y | 3.1 | 1.546E-06 | 9.375E-06 | *** |
| k__Bacteria\| p__Patescibacteria\| c__Saccharimonadia\| o__Saccharimonadales\| f__Unclassified\| g__Unclassified\| s__TM7_phylum | 10~18 y | 3.1 | 6.715E-10 | 6.126E-09 | *** |
| k__Bacteria\|p__Actinobacteriota\|c__Actinobacteria\|o__Bifidobacteriales\|f__Bifidobacteriaceae\|g__Bifidobacterium | <2 y | 3.1 | 0.0065436 | 0.0225413 | * |
| k__Bacteria\|p__Firmicutes\|c__Negativicutes\|o__Veillonellales-Selenomonadales\|f__Selenomonadaceae\|g__Selenomonas | 2~4 y | 3.1 | 2.698E-08 | 1.886E-07 | *** |
| k__Bacteria\|p__Firmicutes\|c__Negativicutes\|o__Veillonellales-Selenomonadales\|f__Veillonellaceae\|g__Megasphaera | 2~4 y | 3.1 | 8.738E-10 | 7.742E-09 | *** |
| k__Bacteria\|p__Firmicutes\|c__Negativicutes\|o__Veillonellales-Selenomonadales\|f__Veillonellaceae\|g__Megasphaera\|s__Megasphaera_micronuciformis | 2~4 y | 3.1 | 8.738E-10 | 7.742E-09 | *** |
| k__Bacteria\| p__Proteobacteria\| c__Gammaproteobacteria\| o__Pasteurellales\| f__Pasteurellaceae\| g__Haemophilus\| s__Haemophilus_sputorum | 10~18 y | 3.1 | 1.619E-06 | 9.79E-06 | *** |
| k__Bacteria\|p__Firmicutes\|c__Clostridia\|o__Lachnospirales\|f__Lachnospiraceae\|g__Oribacterium\|s__Oribacterium_sinus | 2~4 y | 3.1 | 2.121E-09 | 1.729E-08 | *** |
| k__Bacteria\| p__Actinobacteriota\| c__Actinobacteria\| o__Actinomycetales\| f__Actinomycetaceae\| g__Actinomyces\| Unclassified | 10~18 y | 3.1 | 5.503E-10 | 5.129E-09 | *** |
| k__Bacteria\|p__Firmicutes\|c__Negativicutes\|o__Veillonellales-Selenomonadales\|f__Selenomonadaceae\|g__Selenomonas\| | 2~4 y | 3.0 | 1.641E-08 | 1.214E-07 | *** |
| k__Bacteria\|p__Firmicutes\|c__Bacilli\|o__Lactobacillales\|f__Listeriaceae\|g__Listeria | <2 y | 3.0 | 9.508E-05 | 0.0004631 | *** |
| k__Bacteria\|p__Firmicutes\|c__Bacilli\|o__Lactobacillales\|f__Listeriaceae\|g__Listeria\| | <2 y | 3.0 | 9.508E-05 | 0.0004631 | *** |
| k__Bacteria\| p__Bacteroidota\| c__Bacteroidia\| o__Bacteroidales\| f__Porphyromonadaceae\| g__Porphyromonas\| Unclassified | 10~18 y | 3.0 | 7.776E-08 | 5.232E-07 | *** |
| k__Bacteria\|p__Proteobacteria\|c__Gammaproteobacteria\|o__Burkholderiales\|f__Burkholderiaceae\|g__Lautropia | 2~4 y | 3.0 | 1.116E-06 | 6.925E-06 | *** |
| k__Bacteria\| p__Actinobacteriota\| c__Actinobacteria\| o__Actinomycetales\| f__Actinomycetaceae\| g__Actinomyces\| s__Actinomyces_graevenitzii | 10~18 y | 3.0 | 1.408E-11 | 1.713E-10 | *** |
| k__Bacteria\| p__Actinobacteriota\| c__Actinobacteria\| o__Micrococcales\| f__Micrococcaceae\| g__Rothia\| s__Unclassified | 10~18 y | 3.0 | 2.015E-11 | 2.384E-10 | *** |
| k__Bacteria\| p__Firmicutes\| c__Bacilli\| o__Lactobacillales\| f__Streptococcaceae\| g__Streptococcus\| s__Streptococcus_gordonii | 10~18 y | 3.0 | 9.039E-09 | 6.781E-08 | *** |
| k__Bacteria\|p__Firmicutes\|c__Clostridia\|o__Peptostreptococcales-Tissierellales\|f__Family_XI\|g__Finegoldia | <2 y | 3.0 | 0.0029341 | 0.0110245 | * |
| k__Bacteria\| p__Fusobacteriota\| c__Fusobacteriia\| o__Fusobacteriales\| f__Fusobacteriaceae\| g__Fusobacterium\| Unclassified | 10~18 y | 3.0 | 5.273E-12 | 7.519E-11 | *** |
| k__Bacteria\| p__Firmicutes\| c__Negativicutes\| o__Veillonellales-Selenomonadales\| f__Veillonellaceae\| g__Dialister | 10~18 y | 3.0 | 1.688E-10 | 1.674E-09 | *** |
| k__Bacteria\|p__Actinobacteriota\|c__Actinobacteria\|o__Bifidobacteriales\|f__Bifidobacteriaceae\|g__Bifidobacterium\| | <2 y | 3.0 | 0.0046258 | 0.0168801 | * |
| k__Bacteria\|p__Firmicutes\|c__Bacilli\|o__Staphylococcales\|f__Gemellaceae\|g__Gemella\| | <2 y | 3.0 | 0.0005388 | 0.0023626 | ** |
| k__Bacteria\| p__Proteobacteria\| c__Gammaproteobacteria\| o__Burkholderiales\| f__Neisseriaceae\| g__Neisseria\| s__Neisseria_perflava | <2 y | 3.0 | 0.0079267 | 0.0266657 | * |
| k__Bacteria\| p__Actinobacteriota\| c__Actinobacteria\| o__Corynebacteriales\| f__Corynebacteriaceae\| g__Corynebacterium\| s__Corynebacterium_matruchotii | 10~18 y | 3.0 | 3.934E-07 | 2.574E-06 | *** |
| k__Bacteria\|p__Actinobacteriota\|c__Actinobacteria\|o__Propionibacteriales\|f__Propionibacteriaceae\|g__Cutibacterium | <2 y | 3.0 | 3.235E-06 | 1.862E-05 | *** |
| k__Bacteria\|p__Actinobacteriota\|c__Actinobacteria\|o__Propionibacteriales\|f__Propionibacteriaceae\|g__Cutibacterium\| | <2 y | 3.0 | 3.235E-06 | 1.862E-05 | *** |
| k__Bacteria\| p__Firmicutes\| c__Negativicutes\| o__Veillonellales-Selenomonadales\| f__Selenomonadaceae\| g__Selenomonas\| s__Selenomonas_sp. | 10~18 y | 3.0 | 1.914E-08 | 1.397E-07 | *** |
| k__Bacteria\|p__Firmicutes\|c__Bacilli\|o__Lactobacillales\|f__Carnobacteriaceae\|g__Granulicatella | <2 y | 3.0 | 0.0033505 | 0.0125235 | * |
| k__Bacteria\|p__Firmicutes\|c__Bacilli\|o__Lactobacillales\|f__Carnobacteriaceae\|g__Granulicatella\| | <2 y | 3.0 | 0.0015859 | 0.0064791 | ** |
| k__Bacteria\|p__Patescibacteria\|c__Saccharimonadia\|o__Saccharimonadales\|f__unidentified_Saccharimonadales\|g__unidentified_Saccharimonadales | 2~4 y | 3.0 | 1.835E-05 | 9.635E-05 | *** |
| k__Bacteria\| p__Campilobacterota\| c__Campylobacteria\| o__Campylobacterales\| f__Campylobacteraceae\| g__Campylobacter | 10~18 y | 3.0 | 1.519E-12 | 3.55E-11 | *** |
| k__Bacteria\|p__Patescibacteria\|c__Saccharimonadia\|o__Saccharimonadales\|f__unidentified_Saccharimonadales\|g__unidentified_Saccharimonadales\|s__TM7_phylum_sp_oral_clone_FR058 | 2~4 y | 3.0 | 4.973E-05 | 0.0002507 | *** |
| k__Bacteria\| p__Firmicutes\| c__Negativicutes\| o__Veillonellales-Selenomonadales\| f__Veillonellaceae\| g__Dialister\| s__Unclassified | 10~18 y | 3.0 | 8.487E-09 | 6.389E-08 | *** |
| k__Bacteria\| p__Bacteroidota\| c__Bacteroidia\| o__Bacteroidales\| f__Prevotellaceae\| g__Alloprevotella\| s__Alloprevotella_rava | 10~18 y | 3.0 | 2.842E-09 | 2.291E-08 | *** |
| k__Bacteria\|p__Firmicutes\|c__Bacilli\|o__Lactobacillales\|f__Lactobacillaceae\|g__Ligilactobacillus\| | <2 y | 3.0 | 9.508E-05 | 0.0004631 | *** |
| k__Bacteria\| p__Firmicutes\| c__Clostridia\| o__Lachnospirales\| f__Lachnospiraceae\| g__Unclassified | 10~18 y | 3.0 | 6.508E-07 | 4.109E-06 | *** |
| k__Bacteria\| p__Proteobacteria\| c__Gammaproteobacteria\| o__Burkholderiales\| f__Neisseriaceae\| g__Neisseria\| s__Neisseria_elongata | 10~18 y | 3.0 | 9.121E-09 | 6.819E-08 | *** |
| k__Bacteria\| p__Firmicutes\| c__Clostridia\| o__Lachnospirales\| f__Lachnospiraceae\| g__Unclassified\| s__Eubacterium_sp. | 10~18 y | 3.0 | 2.313E-06 | 1.35E-05 | *** |
| k__Bacteria\| p__Bacteroidota\| c__Bacteroidia\| o__Bacteroidales\| f__Prevotellaceae\| g__Prevotella\| s__Prevotella_aurantiaca | 10~18 y | 3.0 | 4.149E-05 | 0.0002117 | *** |
| k__Bacteria\| p__Firmicutes\| c__Clostridia\| o__Clostridia_UCG-014\| f__Unclassified\| g__Unclassified | 10~18 y | 3.0 | 1.62E-10 | 1.622E-09 | *** |
| k__Bacteria\|p__Firmicutes\|c__Bacilli\|o__Lactobacillales\|f__Lactobacillaceae\|g__Ligilactobacillus | <2 y | 3.0 | 0.001762 | 0.0071442 | ** |

**P*<0.05; ***P*<0.01; ****P*<0.001


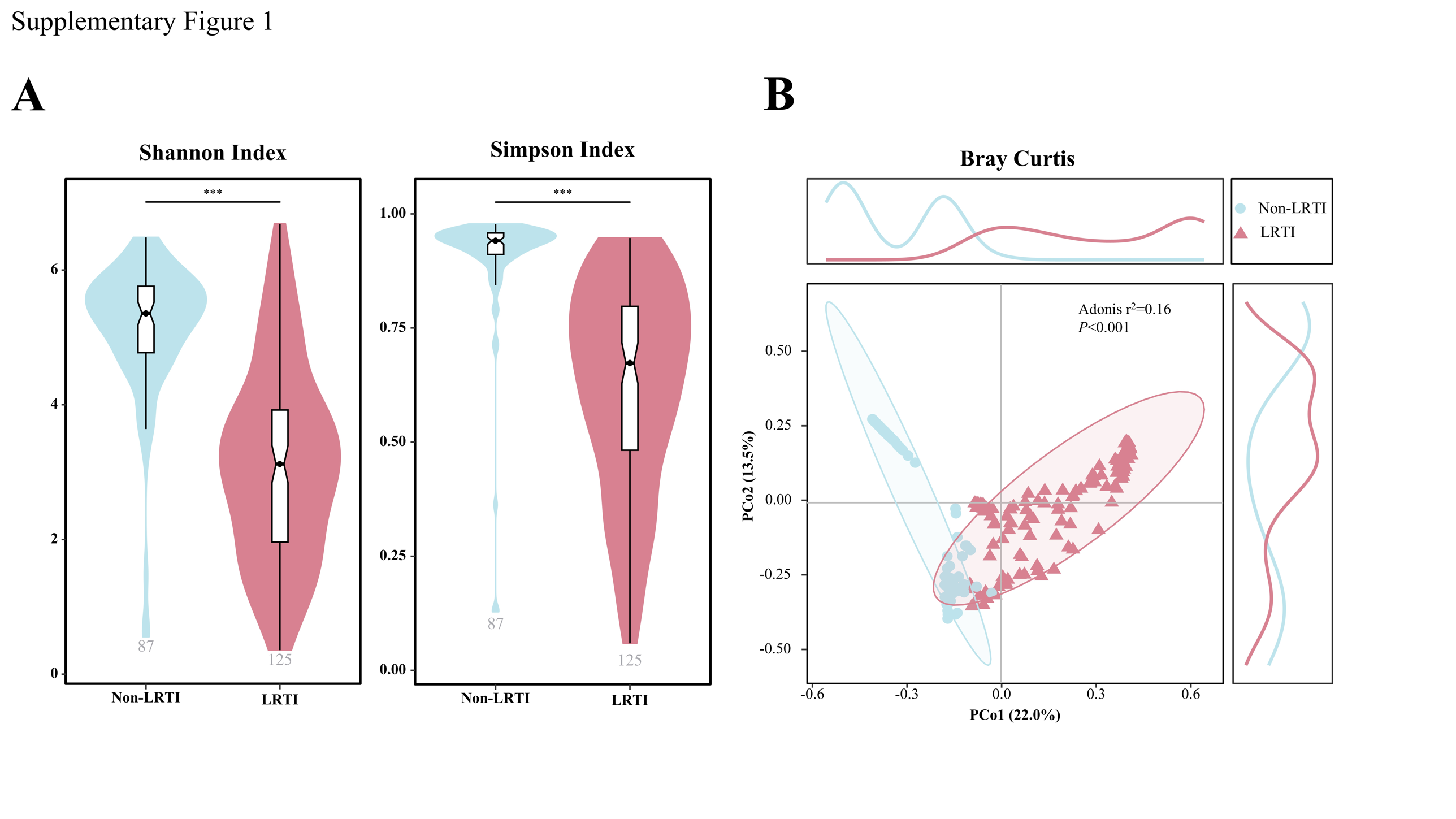


**Figure S1. Comparison of lower respiratory tract microbiota diversity in children with non-LRTI and LRTI.**

(A) Shannon and simpson diversities between non-LRTI and LRTI groups. *P* values were calculated using wilcoxon rank sum test. (B) Principal coordinate analysis (PCoA) based on Bray-Curtis dissimilarities between non-LRTI and LRTI groups. LRTI= lower respiratory tract infections
